# Characteristics and outcomes of patients with acute myeloid leukemia admitted to intensive care unit with acute respiratory failure: a post-hoc analysis of a prospective multicenter study

**DOI:** 10.1186/s13613-023-01172-3

**Published:** 2023-09-02

**Authors:** Carolina Secreto, Dara Chean, Andry van de Louw, Achille Kouatchet, Philippe Bauer, Marco Cerrano, Etienne Lengliné, Colombe Saillard, Laurent Chow-Chine, Anders Perner, Peter Pickkers, Marcio Soares, Jordi Rello, Frédéric Pène, Virginie Lemiale, Michael Darmon, Sofiane Fodil, Ignacio Martin-Loeches, Sangeeta Mehta, Peter Schellongowski, Elie Azoulay, Djamel Mokart

**Affiliations:** 1 Division of Haematology, Department of Oncology, A.O.U. Città Della Salute e della Scienza di Torino, Turin, Italy; 2 Médecine Intensive et Réanimation, APHP, Hôpital Saint Louis, Paris Cité University, Paris, France; 3 Division of Pulmonary and Critical Care, Penn State University College of Medicine, Hershey, PA USA; 4 Department of Medical Intensive Care Medicine, University Hospital of Angers, Angers, France; 5 Pulmonary and Critical Care Medicine, Mayo Clinic, Rochester, MN USA; 6 Hématologie Adulte, Hôpital Saint-Louis, Université Paris Diderot, Paris, France; 7 Hematology Department, Institut Paoli-Calmettes, Marseille, France; 8 Réanimation Polyvalente et Département d’Anesthésie et de Réanimation, Institut Paoli-Calmettes, Marseille, France; 9 Department of Intensive Care, Rigshospitalet, University of Copenhagen, Copenhagen, Denmark; 10 Department of Intensive Care Medicine, Radboud University Medical Center, Nijmegen, The Netherlands; 11 Department of Critical Care and Graduate Program in Translational Medicine, D’Or Institute for Research and Education, Programa de Pós-Graduação Em Clínica Médica, Rio De Janeiro, Brazil; 12 Vall d’Hebron Institute of Research, Barcelona, Spain; 13 CHU Nîmes, Université de Nîmes-Montpellier, Nîmes, France; 14 Medical ICU, Cochin Hospital, Assistance Publique-Hôpitaux de Paris and University Paris Descartes, Paris, France; 15 Medical Intensive Care Unit, APHP, Hôpital Saint-Louis and Paris Diderot Sorbonne University, Paris, France; 16 Department of Intensive Care Medicine, St. James’s Hospital, Dublin, Ireland; 17 Sinai Health System and University of Toronto, Toronto, ON Canada; 18 Department of Medicine I, Medical University of Vienna, Vienna, Austria

**Keywords:** Acute myeloid leukemia, Acute respiratory failure, Intensive care unit, Hospital mortality, Cluster analysis

## Abstract

**Background:**

Acute respiratory failure (ARF) is the leading cause of intensive care unit (ICU) admission in patients with Acute Myeloid Leukemia (AML) and data on prognostic factors affecting short-term outcome are needed.

**Methods:**

This is a post-hoc analysis of a multicenter, international prospective cohort study on immunocompromised patients with ARF admitted to ICU. We evaluated hospital mortality and associated risk factors in patients with AML and ARF; secondly, we aimed to define specific subgroups within our study population through a cluster analysis.

**Results:**

Overall, 201 of 1611 immunocompromised patients with ARF had AML and were included in the analysis. Hospital mortality was 46.8%. Variables independently associated with mortality were ECOG performance status ≥ 2 (OR = 2.79, *p* = 0.04), cough (OR = 2.94, *p* = 0.034), use of vasopressors (OR = 2.79, p = 0.044), leukemia-specific pulmonary involvement [namely leukostasis, pulmonary infiltration by blasts or acute lysis pneumopathy (OR = 4.76, *p* = 0.011)] and liver SOFA score (OR = 1.85, *p* = 0.014). Focal alveolar chest X-ray pattern was associated with survival (OR = 0.13, *p* = 0.001). We identified 3 clusters, that we named on the basis of the most frequently clinical, biological and radiological features found in each cluster: a “leukemic cluster”, with high-risk AML patients with isolated, milder ARF; a “pulmonary cluster”, consisting of symptomatic, highly oxygen-requiring, severe ARF with diffuse radiological findings in heavily immunocompromised patients; a clinical “inflammatory cluster”, including patients with multi-organ failures in addition to ARF. When included in the multivariate analysis, cluster 2 and 3 were independently associated with hospital mortality.

**Conclusions:**

Among AML patients with ARF, factors associated with a worse outcome are related to patient’s background (performance status, leukemic pulmonary involvement), symptoms, radiological findings, the need for vasopressors and the liver SOFA score.

We identified three specific ARF syndromes in AML patients, which showed a prognostic significance and could guide clinicians to optimize management strategies.

**Supplementary Information:**

The online version contains supplementary material available at 10.1186/s13613-023-01172-3.

## Introduction

Despite the improvement in survival [1–4], many patients with Acute Myeloid Leukemia (AML) still experience acute complications, related to the disease itself or to the anti-leukemic treatment, and will require intensive care unit (ICU) management.

Acute respiratory failure (ARF) is the leading cause of ICU admission in patients with AML [5] and its management is challenging, because of the unique features that ARF could display in this context [6–8]. In recent years, an increasing number of immunocompromised patients are being admitted to ICUs, with improved survival compared to the past [9–11], and a relevant proportion of them is represented by AML patients with ARF who need prompt critical management [12–14]. Moreover, these patients show a complex type of immunosuppression, depending on the disease phase and on the treatment administered, and these factors are to consider in their management; at diagnosis, AML immunosuppression is mostly related to the hematological malignancy (e.g., hyperleukocytosis with functional neutropenia) [15], while, after hematopoietic stem cell transplantation, immunosuppression is mainly related to cellular immune-modulation and immune-reconstitution [16].

Data on prognostic factors affecting short-term outcome are needed to maximize cure intensity in patients that will actually benefit from it, but studies focusing on this subset of patients are scarce, mostly based on retrospective data and small cohorts.

We therefore analyzed a homogeneous cohort of AML patients with ARF, to investigate potential risk factors for post-ICU hospital mortality; we also aimed to determine whether specific subgroups of AML patients were recognizable among the study population.

## Patients and methods

This is a post-hoc analysis of the EFRAIM study, a multicenter, international prospective cohort study on onco-hematological patients with hypoxemic ARF, admitted to 68 ICUs experienced in the management of critically ill immunocompromised patients [17].

### Study population

In this subgroup analysis, we evaluated all the patients with AML admitted to the participating ICUs between November 2015 and July 2016 for acute hypoxemic ARF (defined as breathing difficulty with the use of accessory muscles of respiration, respiratory distress, dyspnea at rest or cyanosis, or PaO_2_ < 60 mmHg or SpO_2_ < 90% on room air, or tachypnea > 30/min, onset of respiratory symptoms < 72 h and the need for oxygen ≥ 6 L/min).

The full protocol describing inclusion/exclusion criteria, as well as data collection, has been previously published [17]  and is resumed in Additional file 1.

Participating providers obtained institutional review board (IRB) approval from their institutions, in accordance with local ethics regulations.

The primary outcome of this study was to evaluate all-cause mortality at hospital discharge; the secondary outcome was to identify specific subgroups of AML patients with ARF.

### Statistical analysis

Data are presented as numbers (percentages) for qualitative variables and as median (25th-75th percentiles) or mean [standard deviations (SD)] for quantitative variables. Data were firstly compared between survivors and non-survivors at hospital discharge, using the Mann–Whitney test for continuous variables and the Chi-Square or Fisher’s exact tests for categorical variables. A *p* < 0·05 was considered statistically significant. We performed a multivariate logistic regression analysis to identify independent variables associated with hospital mortality, as measured by the estimated odds ratio (OR) and 95% confidence interval (95% CI). Factors with significance or borderline significance (*p* < 0·1) in the univariate analyses and factors described as relevant in previous studies were then included in a multivariable regression model with backward stepwise variable selection. The required significance level was set at a *p* < 0·05. The Hosmer–Lemeshow test was used to check goodness-of-fit of the selected logistic model. Similarly, for patients presenting with cough at ICU admission, we performed a multivariate analysis using logistic regression to identify diagnoses independently associated with this symptom among the 11 etiologies involved in the ARF onset. The same approach was used for patients with a focal alveolar image on chest X-ray.

### Cluster determination

Secondly, we aimed to identify different subgroups of patients based on their baseline clinical, biological and radiological parameters, as well as the therapeutic approach applied. This purpose was achieved through a multi-step strategy:

A) Firstly, we performed a factor analysis of mixed data (FAMD), a principal component method dedicated to analyzing complex datasets containing both quantitative and qualitative variables [18]. This dimensions’ reduction algorithm summarizes the main variances of the variables and transposes them on lower dimensional planes, which allows simple visual evaluation of the data in a compact format; furthermore, it reduces the background noise of the variables and allows subsequent analysis, such as clustering. Therefore, we reduced the wide dimensionality of the original data to fewer and balanced latent dimensions.
B) Cluster analysis is one of the most popular unsupervised learning methods to identify subgroups sharing similar characteristics, with no need for predefined information; we thus performed an ascendant hierarchical cluster analysis (AHCA) on the dimensions previously provided by the FAMD model, using an ascendant algorithm on the Euclidean distances between points and according to the Ward’s method; it allowed to minimize the total intracluster variance and to generate the dendrogram [19], whose visual inspection permitted the identification of the optimal number of clusters.
C) Thirdly, comparisons between the clusters were assessed using the Chi-square for qualitative variables and the Kruskal–Wallis test for quantitative variables.

Clusters’ names were defined based on the most frequently clinical, biological and radiological situations found in each cluster, in order to summarize and label each group of patients at one glance.

Details on clustering statistical methods are provided in Additional file 1.

Each cluster was then included as qualitative variable in the first logistic regression model to assess its impact on hospital mortality. Cumulative incidence curves were used to describe cumulative incidence of hospital death and comparisons between groups were performed using Gray’s test. All tests were two-sided, and *p* < 0.05 was considered statistically significant. All statistical analyses were performed within R-3·4·9 environment, we used FactoMineR, factoextra and missMDA packages for FAMD [20, 21].

## Results

### Study population

**Fig. 1:**
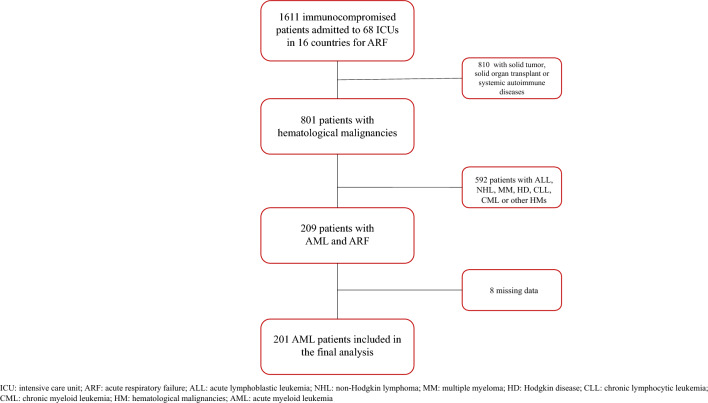
Study flowchart

Overall, 201 of the 1611 immunocompromised patients with ARF had AML and were included in the final analysis (Fig. 1).

Patients’ characteristics are displayed in Table 1. Median age was 60·3 years (IQR 50.5–68.1) and 112 patients (55.7%) were admitted to ICU at the initial phase of the disease, namely at diagnosis [83 (41.3%)] or during the first line of treatment [29 (14.4%)]. Fifty-eight patients (28.9%) had received hematopoietic stem cell transplantation (HSCT) and one in 4 patients [52 (25.9%)] was severely neutropenic. Data on AML treatment were available in 54 patients (26.9%,); among them, 55.6% had received intensive chemotherapy, 9.3% low-intensity treatment (e.g., hypomethylating agents), and 33.3% was receiving post-HSCT immunosuppressive therapy at the time of ICU admission.

**Table 1:** Patients’ characteristics at ICU admission (univariate analysis)

Characteristic	Missing data, n (%)	Overall (n = 201)	Alive (n = 107)	Dead (n = 94)	*p* value
Age, years, median (IQR)		60·3 (50.5–68.1)	57.1 (44.9–66.9)	62.7 (53.0–68.7)	0.07
Male sex, n (%)		119 (59.2)	64 (59.8)	55 (58.5)	0.89
Disease status, n (%)*					**0.07**
New diagnosis		83 (41.3)	41 (38.3)	42 (44.7)	
First line of treatment		29 (14.4)	22 (20.6)	7 (7.4)	
≥ 2 line of treatment		29 (14.4)	11 (10.3)	18 (19.1)	
Remission		25 (12.4)	15 (14.0)	10 (10.6)	
Active disease		7 (3.5)	3 (2.8)	4 (4.3)	
Unknown		28 (13.9)	15 (14.0)	13 (13.8)	
Transplant, n (%)					**0.09**
HSCT		58 (28.9)	26 (24.3)	32 (34.0)	
ASCT		2 (1.0)	0 (0.0)	2 (2.1)	
No HSCT		141 (70.1)	81 (75.7)	60 (63.8)	
Non-hematological systemic diseases, n (%)		12 (6.0)	8 (7.5)	4 (4.3)	0.51
ECOG ≥ 2, n (%)		67 (39.2)	28 (31.1)	39 (48.1)	0.03
Comorbidities, n (%)					
Cardiac	14 (7.0)	46 (24.6)	25 (24.5)	21 (24.7)	1.00
COPD	10 (5.0)	20 (10.5)	13 (13.0)	7 (7.7)	0.34
Diabetes	9 (4.5)	25 (13.0)	11 (11.0)	14 (15.2)	0.51
Chronic Renal Failure	6 (3.0)	11 (5.6)	6 (5.8)	5 (5.5)	1.00
Cirrhosis	7 (3.5)	2 (1.0)	2 (1.9)	0 (0.0)	0.53
Prophylaxis, n (%)					
Antifungal		131 (65.2)	67 (62.6)	64 (68.1)	0.68
Viral		80 (39.8)	35 (32.7)	45 (47.9)	0.09
Pneumocystis		41 (20.4)	18 (16.8)	23 (24.5)	0.40
Code status at ICU admission, n (%)					**0.02**
Full code		173 (86.1)	98 (91.6)	75 (79.8)	
Time-limited trial		4 (2.0)	0 (0.0)	4 (4.3)	
Early admission		5 (2.5)	0 (0.0)	5 (5.3)	
DNI/DNR		8 (4.0)	4 (3.7)	4 (4.3)	
Unknown		11 (5.5)	5 (4.6)	6 (6.4)	
Clinical features at admission and day 1, n (%)					
Chest pain	17 (8.5)	28 (15.2)	19 (19.4)	9 (10.5)	0.14
Cough	15 (7.5)	79 (42.5)	33 (33.3)	46 (52.9)	0.01
Sputum	16 (8.0)	26 (14.1)	8 (8.2)	18 (20.7)	0.03
Hemoptysis	13 (6.5)	20 (10.6)	10 (10.1)	10 (11.2)	0.99
Muscle pain	15 (7.5)	9 (4.8)	4 (4.0)	5 (5.7)	0.84
Rhinorrhea	17 (8.5)	4 (2.1)	2 (2.0)	2 (2.2)	1.00
Rash	13 (6.5)	20 (10.6)	12 (12.1)	8 (9.0)	0.65
Neutropenia		52 (25.9)	29 (27.1)	23 (24.5)	0.79
White blood cells, × 10^9^/L, median (IQR)		4.00 (0.00–26.00)	3·00 (0.00–24.50)	6.00 (0.00–25.50)	0.46
Platelets, median (IQR)		28.00 (13.00–64.50)	32.50 (15.00–68.75)	23.00 (13.00–48.00)	0.07
Temperature, median (IQR)		38.4 (37.4–39.3)	38.5 (37·4–39.5)	38.4 (37.1–39.1)	0.21
RR		33 (27–38)	32.5 (27–38)	33 (27–39)	0.68
SpO2		92 (89–95)	92 (89–95)	92 (89–95)	0.43
PaO2, mmHg		68.0 (58.0–85.2)	65.5 (54.5–83.7)	68.5 (58.2–88.7)	0.31
PaCO2, mmHg		36.5 (31.0–47.2)	35.0 (30.0–43.5)	39.0 (31.0–50.0)	0.08
PaO2/FiO2		150 (98–232)	157.5 (94–255.5)	137 (101–210)	0.44
Berlin category (patients with ARDS), n (%)		145 (72.1)	73 (68.2)	72 (76.6)	0.21
*Mild*		32 (22.1)	17 (23.3)	15 (20.8)	0.55
*Moderate*		70 (48.3)	32 (43.8)	38 (52.8)	
*Severe*		43 (29.7)	24 (32.9)	19 (26.4)	
Oxygen Flow, L/min, median (IQR)		11·5 (8–15)	11 (7–15)	11 (9–13.5)	0.58
SOFA score at admission, median (IQR)					
Respiratory		2 (2–3)	2 (1–3)	2 (2–3)	0.36
Cardiovascular		1 (0–4)	0 (0–3)	1 (0–4)	0.06
Hepatic		0 (0–1)	0 (0–1)	1 (0–2)	0.01
Neurological		0 (0–1)	0 (0–0)	0 (0–1)	0.24
Renal		0 (0–1)	0 (0–1)	0 (0–2)	0.19
Hemostatic		3 (2–4)	3 (2–4)	3 (3–4)	0.06
Total		8 (6–12)	7 (5–10)	9 (7–13)	0.001
Days from ARF to ICU admission, median (IQR)		1 (0–3)	1 (0–2)	1 (0–3.25)	0.21
Days from AML diagnosis to ICU admission, median (IQR)		22.50 (2–165)	24 (4–173)	20 (0.50–160.5)	0.37
Radiological findings					
*Chest X-ray pattern, n (%)*					
Alveolar Focal		39 (19.4)	29 (27.1)	10 (10.6)	0.01
Diffuse		77 (38.3)	36 (33.6)	41 (43.6)	0.19
Interstitial Focal		10 (5.0)	9 (8.4)	1 (1.1)	0.04
Diffuse		100 (49.8)	45 (42.1)	55 (58.5)	0.03
Pleural effusion		78 (39.0)	44 (41.5)	34 (36.2)	0.53
Fibrosis		5 (2.5)	1 (0.9)	4 (4.3)	0.29
N.quadrants involved, median (IQR)		3 (2–4)	3 (2–4)	4 (2–4)	0.02
*CT-scan patterns, n (%)*					
Halo sign		2 (1.0)	1 (0.9)	1 (1.1)	1.00
Nodules		14 (7.0)	10 (9.3)	4 (4.3)	0.26
Pleural effusion		61 (30.5)	31 (29.2)	30 (31.9)	0.80
Alveolar consolidation		68 (33.8)	32 (30.0)	36 (38.3)	0.21
Focal		35 (17.4)	20 (18.7)	15 (16.0)	0.71
Diffuse		33 (16.4)	12 (11.2)	21 (22.3)	0.04
Ground glass opacities		59 (29.4)	30 (28.0)	29 (31)	0.66
Focal		19 (9.5)	15 (14.0)	4 (4.3)	0.03
Diffuse		40 (20.0)	15 (14.0)	25 (26.6)	0.03
Septal thickening		22 (11.0)	11 (10.4)	11 (11.7)	0.94

*HSCT* hematopoietic stem cell transplantation; *ASCT* autologous stem cell transplantation; *ECOG* Eastern Cooperative Group Performance Status; *DNI/DNR* Do Not Intubate/Do Not Resuscitate; *RR* respiratory rate

^*^New diagnosis: newly diagnosed AML who are about to start the appropriate treatments; active disease: already diagnosed AML with active and uncontrolled disease, off-therapy after the failure of previous treatments (e.g., palliative setting)

In univariate analysis, respiratory values at admission and during the first day of ICU stay (namely SpO_2_, PaO_2_, PaO_2_/FiO_2_ ratio and oxygen flow) were non significantly different in patients alive or dead at hospital discharge.

The median time from ARF onset to ICU admission was short (1 day, IQR 0–3).

### Diagnostic workup, ARF etiology and ICU support

Extensive diagnostic workup (Additional file 2) was carried out to determine ARF etiology. This was identified in 173 patients (86·1%), with infection as the most common cause [103 (51.2%)], mainly due to bacteria [58 (56.3%)] and viruses [26 (25%)].

Leukemia-specific pulmonary involvement was found in 35 patients (17.4%), representing the most common cause of non-infectious etiology.

Management strategies at admission and during ICU stay are shown in Table 2. Notably, 61 patients (30.3%) received AML-specific chemotherapy in ICU, particularly those with newly diagnosed AML (39 patients, 64% of patients receiving chemotherapy).

**Table 2:** ARF-associated organ dysfunction and treatments during ICU stay (univariate analysis)

Characteristic	Overall (n = 201)	Alive (n = 107)	Dead (n = 94)	*p* value
Septic shock, n (%)	98 (48.8)	41 (38.3)	57 (60.6)	0.003
Liver dysfunction, n (%)	55 (27.4)	20 (18.7)	35 (37.2)	0.005
Coma/CNS, n (%)	52 (25.9)	26 (24.3)	26 (27.7)	0.70
First-line ventilation strategy at ICU admission, n (%)				
Standard oxygen	101 (50.2)	58 (54.2)	43 (45.7)	0.26
HFNC	72 (35.8)	45 (42.1)	27 (28.7)	0.06
NIV	65 (32.3)	31 (29)	34 (36.2)	0.29
IMV	76 (37.8)	33 (30.8)	43 (45.7)	0.04
Overall ventilation strategy during ICU stay, n (%)				
HFNC	127 (63.2)	75 (70.1)	52 (55.3)	0.04
NIV	80 (39.8)	40 (37.4)	40 (42.6)	0.55
IMV	120 (59.7)	54 (50.5)	66 (70.2)	0.007
Days from ICU admission to IMV, median (IQR)	0 (0–2)	0 (0–2)	0.50 (0–2)	0.74
ICU supportive treatments				
Volume expansion, ml, median (IQR)	1500 (500–2725)	1159 (250–2500)	1590 (857–3000)	0.10
Vasopressor use, n (%)	119 (59.2)	51 (47.7)	68 (72.3)	0.001
RRT, n (%)	44 (21.9)	18 (16.8)	26 (27.7)	0.09
Chemotherapy, n (%)	61 (30.3)	31 (29.0)	30 (31.9)	0.77
Steroids, n (%)	49 (24.4)	24 (22.4)	25 (26.6)	0.60
Immunosuppressant drugs, n (%)	59 (29.4)	25 (23.4)	34 (36.2)	0.07
Anti-infectious agents, n (%)	179 (89)	94 (46.8)	85 (42.3)	0.65
Transfusion, n (%)				
Red blood cells	2 (1–4)	2 (1–4)	2 (0–4)	0·98
Platelets	3 (1–5.50)	3 (1–5)	3 (1–6)	0·92
Plasma	0	0	0	0·69
Complications in ICU, n (%)				
VAP	22 (11.3)	11 (10.6)	11 (12.1)	0.92
Drug-related toxicity	25 (13.0)	12 (11.7)	13 (14.6)	0.70
MDR infections	33 (17.2)	16 (15.5)	17 (19.1)	0.64
Cardiac arrest at intubation	3 (1·7)	0	3 (3.5)	0.23

*CNS* central nervous system; *HFNC* high-flow nasal cannula; *NIV* noninvasive ventilation; *IMV* invasive mechanical ventilation; *RRT* renal replacement therapy; *VAP* ventilator-associated pneumonia; *MDR* multidrug resistant

Overall oxygenation strategy during ICU stay was high-flow nasal cannula (HFNC) in 127 patients (63.2%), non-invasive ventilation (NIV) in 80 patients (39.8%) and invasive mechanical ventilation (IMV) in 120 patients (59.7%). During the first 24 h of ICU stay, the most invasive oxygenation strategy was IMV in 75 patients (37.3%), NIV in 49 (24.4%), HFNC in 44 (21.9%) and standard oxygen only in 33 (16.4%). The median time from ICU admission to IMV was 0 days (IQR 0–2). Among invasively mechanically ventilated patients, 22 (18.3%) developed ventilator-associated pneumonia (VAP).

### Hospital mortality

ICU, hospital, and day-90 mortality rates were 33.8% (68 deaths), 46.8% (94 deaths), and 59.3% (108 deaths, 19 missing information), respectively.

In univariate analysis, factors significantly associated with hospital mortality were ECOG performance status ≥ 2 (*p* = 0.03); patient’s code status on ICU admission [recorded as full code management, time-limited trial, early admission, do not intubate (DNI)/ do not resuscitate (DNR), or unknown] (*p* = 0.02); cough (*p* = 0.01); sputum production (*p* = 0.03); a higher liver and total SOFA score (*p* = 0.01 and *p* = 0.001 respectively); a diffuse interstitial pattern on chest X-ray (*p* = 0.03) with a higher number of X-ray quadrants involved (*p* = 0.02), as well as diffuse alveolar and ground glass patterns on computerized tomography (CT) scan (*p* = 0.04 and *p* = 0.03); shock and use of vasopressors (*p* = 0.03 and *p* = 0.001 respectively); liver failure (*p* = 0.005) and IMV (*p* = 0.007). Conversely, the main factors associated with survival were focal alveolar or interstitial pattern on chest X-ray (*p* = 0.006 and *p* = 0.04), focal ground glass opacities on CT-scan (*p* = 0.03) and HFNC as oxygenation strategy (*p* = 0.04). Neither neutropenia nor ARF etiology were associated with outcome.

By multivariate analysis, ECOG Performance Status ≥ 2 (OR = 2.79, 95% CI 1·05–7.43, *p* = 0.04), cough (OR = 2.94, 95% CI 1.09–7.96, *p* = 0.03), use of vasopressors (OR = 2.79, 95% CI 1.03–7.60, *p* = 0.04), leukemia-specific pulmonary involvement (OR = 4.76, 95% CI 1.44–7.80, *p* = 0.01) and liver SOFA score (OR = 1.85, 95% CI 1.13–3.02, *p* = 0.01) were associated with hospital mortality. In contrast, focal alveolar chest X-ray pattern was associated with survival (OR = 0.13, 95% CI 0.04–0.45, *p* = 0.001). At ICU admission, viral infection was the only diagnosis independently associated with the presence of cough (OR = 3.15, 95% CI 1·28–7.78, p = 0.01), while microbiologically documented gram-negative pulmonary sepsis was the only diagnosis independently associated with the presence of an alveolar focal chest X-ray (OR = 4.62, 95% CI 1.71–12.50, p = 0.003).

### FAMD and comparison between clusters

**Fig. 2:**
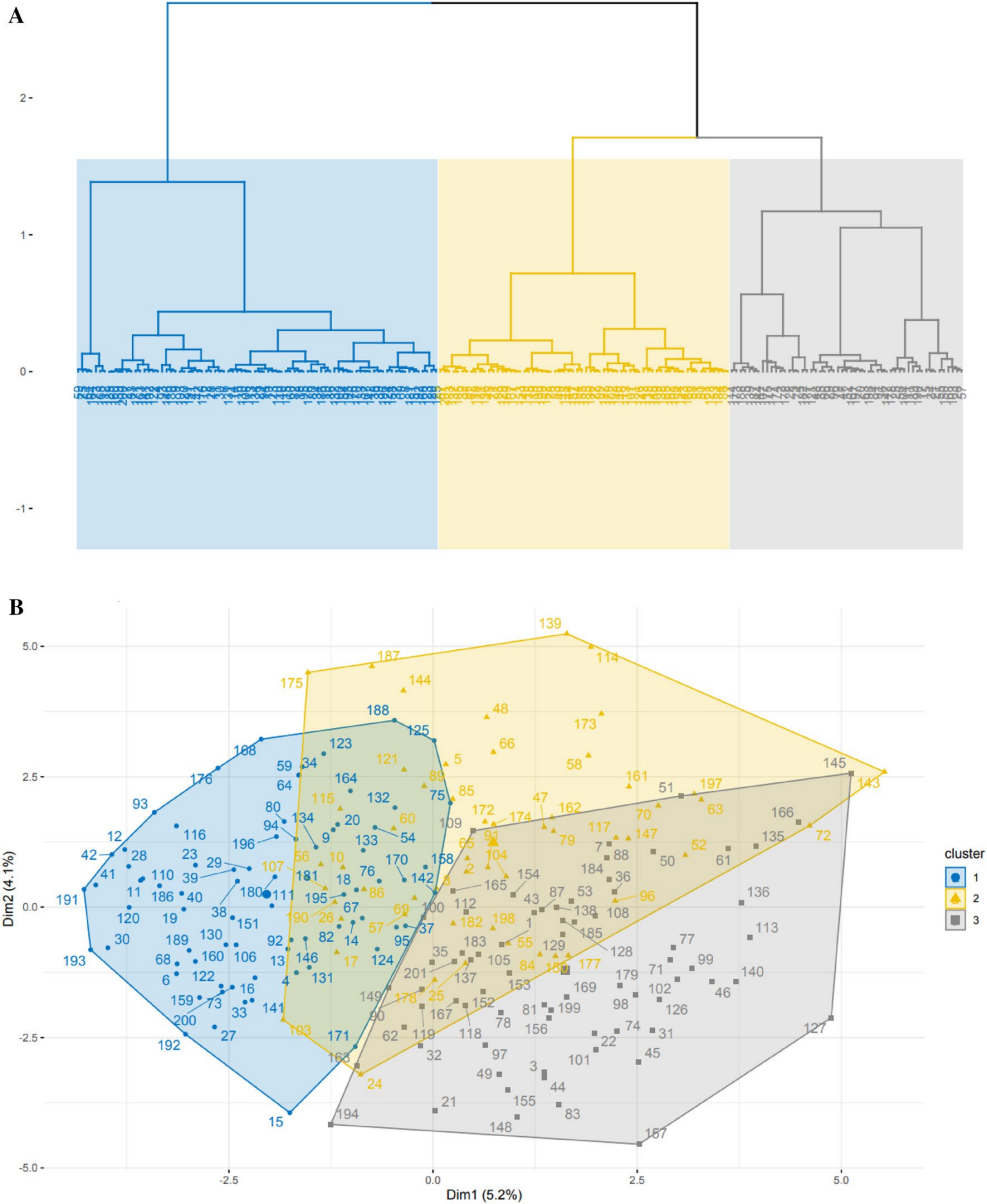
**A** dendrogram showing the hierarchical cluster analysis forming 3 clusters. **B** scatterplot showing the 201 patients’ distribution in the 3 clusters, based on the first two dimensions obtained from the factor analysis of mixed data (FAMD) model

Three clusters of patients were identified through the FAMD and the AHCA (Fig. 2, Additional files 3, 4 and 5). Details on cluster’ definitions are provided in Additional files 1 and 6.

The comparison between the main variables of each cluster (Table 3), significantly correlated with clusters determination after AHCA, provides insights into cluster definition at the patient’s bedside. Patients’ general characteristics (e.g., age, sex, ECOG performance status, comorbidities, and disease status at ICU admission) did not differ between the 3 clusters.

**Table 3:** Comparison of the significant variables between cluster 1, 2 and 3

Characteristic	Clusters			*p* value
	1 (n = 76)	2 (n = 54)	3 (n = 71)	
Transplant, n (%)				** < 0.001**
No	67 (88.2)	26 (48.1)	48 (67.6)	
ASCT	0	0	2 (2.8)	
HSCT	9 (11.8)	28 (51.9)	21 (29.6)	
Cinical features, n (%)				
Chest pain	15 (22.1)	11 (22.0)	2 (3.0)	0.003
Cough	21 (30.4)	35 (68.6)	23 (34.8)	< 0.001
Sputum	8 (11.4)	13 (26.0)	5 (7.7)	0.01
Rash	5 (7.1)	10 (20.0)	5 (7.4)	0.04
Neutropenia	14 (18.4)	11 (20.4)	27 (38·0)	0.01
Berlin category (patients with ARDS), n (%)	44 (57.9)	47 (87.0)	55 (77.5)	0.006
* Mild*	17 (38.6)	5 (10.9)	10 (18.2)	
* Moderate*	21 (47.7)	25 (54.3)	24 (43.6)	
* Severe*	6 (13.6)	16 (34.8)	21 (38.2)	
Radiological findings, n (%)				
Diffuse interstitial chest X-ray pattern	38 (50.0)	35 (64.8)	27 (38.0)	0.01
Septal thickening on CT scan	0	14 (25.9)	8 (11.4)	< 0.001
Pleural effusion on CT scan	13 (17.1)	28 (51.9)	20 (28.6)	< 0.001
Nodules on CT scan				< 0.001
* None*	73 (96.1)	31 (57.4)	66 (93.0)	
* Centrolobular*	2 (2.6)	11 (20.4)	1 (1.4)	
* Diffuse*	1 (1.3)	12 (22.2)	4 (5.6)	
Alveolar consolidation on CT scan				< 0.001
* None*	61 (80.3)	18 (33.3)	54 (76.1)	
* Focal*	8 (10.5)	18 (33.3)	9 (12.7)	
* Diffuse*	7 (9.2)	18 (33.3)	8 (11.3)	
Ground glass opacities on CT scan				< 0.001
* None*	67 (88.2)	19 (35.2)	56 (78.9)	
* Focal*	3 (3.9)	13 (24.1)	3 (4.2)	
* Diffuse*	6 (7.9)	22 (40·.7)	12 (16.9)	
N. X-ray quadrants involved, median (IQR)	2 (0.00–4.00)	3 (1.25–4.00)	1 (0.00–3.00)	0.007
ARF etiology, n (%)				
Extrapulmonary	5 (6.6)	1 (1.9)	16 (22.5)	< 0.001
Leukemia-specific pulmonary involvement	20 (26.3)	6 (11.1)	9 (12.7)	0.03
Bacterial				0.02
* Clinically documented*	8 (10.5)	10 (18.5)	7 (9.9)	
* Micriobiologically documented*	8 (10.5)	15 (27.8)	10 (14.1)	
* Non bacterial*	60 (78.9)	29 (53.7)	54 (76.1)	
Documented GNB infection	3 (3.9)	11 (20.4)	19 (26.8)	0.001
Viral	4 (5.3)	13 (24.1)	9 (12.7)	0.007
Known ARF etiology at ICU admission	42 (60.0)	20 (37.0)	31 (46.3)	0.04
Organ dysfunction and treatments, n (%)				
IMV	25 (32.9)	39 (72.2)	56 (78.9)	< 0.001
HFNC	42 (55.3)	46 (85.2)	39 (54.9)	< 0.001
O_2_ flow > 10 L/min	8 (18.6)	10 (55.6)	10 (35.7)	0.02
Vasopressor use	18 (23.7)	34 (63.0)	67 (94.4)	< 0.001
Septic shock	13 (17.1)	24 (44·4)	61 (85.9)	< 0.001
RRT	6 (7.9)	8 (14.8)	30 (42.3)	< 0.001
Chemotherapy	32 (42.1)	11 (20.4)	18 (25.4)	0.02
Liver dysfunction	14 (18.4)	10 (18.5)	31 (43.7)	0.001
Coma/CNS	10 (13.2)	10 (18.5)	32 (45.1)	< 0.001
Appropriate ATB at ICU admission				0.01
* No*	4 (5.3)	4 (7.4)	1 (1.4)	
* Early*	25 (32.9)	30 (55.6)	41 (57.7)	
* Later*	30 (39.5)	10 (18.5)	16 (22.5)	
* Never*	10 (13.2)	6 (11.1)	12 (16.9)	
* Unknown*	7 (9.2)	4 (7.4)	1 (1.4)	
Ventilator-associated pneumonia	7 (9.5)	11 (21.2)	4 (5.8)	0.03
SOFA score and respiratory variables at ICU admission, median (IQR)				
Respiratory	2 (0.25–2.00)	3 (2.00–3.50)	3 (2.00–3.75)	< 0.001
Cardiovascular	0 (0.00–1.00)	0 (0.00–1.00)	4 (0.50–4.00)	< 0.001
Hepatic	0 (0.00–1.00)	0 (0.00–1.00)	1 (0.00–2.00)	< 0.001
Hemostatic	3 (2.00–4.00)	3 (2.00–4.00)	4 (3.00–4.00)	0.02
Neurological	0 (0.00–0.00)	0 (0.00–0.00)	0 (0.00–2.00)	< 0.001
Renal	0 (0.00–1.00)	0 (0.00–1.00)	1 (0.00–2.00)	0.001
Total SOFA	7 (4.00–8.00)	8 (6.00–9.00)	12 (10.00–14.00)	< 0.001
Total SOFA without respiratory SOFA	5 (3.00–6.00)	5 (3.00–7.00)	10 (7.25–11.00)	< 0.001
RR, median (IQR)	31·5 (25.00–36.25)	35 (28.25–40.75)	30.5 (27.00–40.00)	0.08
SpO_2_, median (IQR)	94 (91.00–96.75)	90 (87.00–93.00)	91.5 (88.00–94.75)	< 0.001
PaCO_2_, median (IQR)	33 (30.00–38.00)	40 (34.50–47.50)	41.00 [31.75–51.25)	0.003
O_2_ flow, median (IQR)	5·00 [3.00–10.00]	12.00 (8.00–15.00)	10 (6.00–15.00)	0.007
PaO_2_/FiO_2_, median (IQR)	210 (130.00–284.00)	114.5 (87.25–187.00)	125 (89.25–194.00)	< 0.001
White blood cells, × 10^9^/L, median (IQR)	9 (1.00–48.00)	3 (1.00–10.00)	1 (0.00–14.00)	0.005
Volume expansion, median (IQR)	1018 (162.25–2000.00)	1130.5 (347.50–2344.00)	2000 (1137.50–3684.00)	0.001
Plasma, median (IQR)	0 (0.00–0.00)	0 (0.00–0.00)	0 (0.00–0.00)	0.03
Platelets, median (IQR)	2 (1.00–4.00)	2 (0.00–5.00)	4 (1.00–6.25)	0.04
Days from AML diagnosis to ICU admission, median (IQR)	9.5 (0.00–48.00)	69 (9.00–437.00)	43 (7.00–157.00)	0.002
Days from ICU admission to IMV, median (IQR)	1 (0.00–2.00)	0 (0.00–2.00)	0 (0.00–1.00)	0.04
Days from ARF to ICU admission, median (IQR)	1 (0.00–2.00)	2 (1.00–6.00)	0 (0.00–2.00)	0.001
Time from ARF to ICU admission > 3 days, n (%)	15 (20.8)	18 (34.0)	11 (15.7)	0.05

*HSCT* hematopoietic stem cell transplantation; *ASCT* autologous stem cell transplantation; *GNB* Gram-negative bacteria; *IMV* invasive mechanical ventilation; *HFNC* high-flow nasal cannula; *RRT* renal replacement therapy; *CNS* central nervous system; *ATB* antibiotic therapy; *RR* respiratory rate

#### Cluster 1 (the leukemic cluster)

In the first cluster (n = 76), ARF severity seemed to be milder, as highlighted by the higher values of SpO2 (94% vs 90% and 92%) and the lower respiratory SOFA (median 2 vs 3 and 3) compared to cluster 2 and 3, with 67% of patients who finally did not require IMV; indeed, patients in cluster 1 had lower oxygen needs at ICU admission (< 10L in 81%). Furthermore, most patients in cluster 1 had fewer organ failures in addition to ARF and were less symptomatic at ICU admission. Patients showed higher leukocytosis compared to the overall population (mean WBC 52 × 10^9^/L in cluster 1 vs 14 × 10^9^/L in cluster 2 and 26 × 10^9^/L in cluster 3), and they more frequently received chemotherapy in the ICU (42% vs 20% and 25%); time from AML diagnosis to ICU admission was shorter (median 9.5 days). Leukemia-specific pulmonary involvement was found in 26% of the patients, more frequently than in clusters 2 and 3 (11% and 13%, respectively).

#### Cluster 2 (the pulmonary cluster)

Patients in cluster 2 (n = 54) presented more respiratory symptoms, especially cough (69% vs 30% and 39%); furthermore, they had a trend to a higher respiratory rate (median 35 vs 32 and 31) and a lower SpO2 (median 90% vs 94% and 92%). Radiological findings played an important role in defining the cluster: overall, these patients had more defined chest X-ray and CT patterns (both alveolar and ground glass) compared to the overall cohort and, among them, diffuse pulmonary involvements were the most represented. Indeed, patients presented more diffused interstitial chest X-ray pattern (65% vs 50% and 38%), more lung quadrants involvement (median 3 vs 2 and 1), more diffuse alveolar consolidations (33% vs 11% and 13%), more diffuse ground glass opacities (41% vs 8% and 17%) and pleural effusion (52% vs 17% and 29%); conversely, the respiratory SOFA was similar to the one of the rest of the population (median 3 vs 2 and 3). The time elapsed between ARF onset and ICU admission was significantly longer compared to cluster 1 and 3 (median 2 days vs 1 and 0); indeed 34% of the patients were admitted to ICU at > 3 days from ARF onset. Infections were the more frequent etiologies of ARF, namely bacterial (46% vs 21% and 24%) and viral infections (24% vs 5% and 13%).

The lung injury was severe, as more patients needed HFNC (85% vs 55% and 55%) and only 28% were finally not intubated, with a very short delay between ICU admission and IMV. The rate of VAP was also higher (20% vs 10% and 6%). This cluster included more heavily immunocompromised patients compared to cluster 1 and 3, namely HSCT patients (52% vs 12% and 30%); indeed, time from AML diagnosis to ICU admission was longer (median 69 days vs 9·5 and 43). Moreover, these patients received steroids more frequently (33% vs 15% and 20%).

#### Cluster 3 (the clinical inflammatory cluster)

In the third cluster (n = 71), the leading feature was the presence of organ failures in addition to ARF; indeed, the majority of patients presented with septic shock (86% vs 17% and 44%), required higher volume of crystalloids (median 2000 ml vs 1018 ml in cluster 1 and 1130 ml in cluster 2) and vasopressors (94% vs 24% in cluster 1 and 63% in cluster 2); moreover, renal replacement therapy (42% vs 8% and 15%), neurological complications (45% vs 13% and 19%) and liver failure (44% vs 18% and 19%) occurred more frequently compared to cluster 1 and 2. Indeed, the total SOFA score was higher (median 12 vs 7 and 8), as well as the SOFA scores of each single organ. A high percentage of patients needed IMV (79% vs 33% and 72%), even if the delay between ARF onset and ICU admission was very short (median 0 days vs 1 and 2) and the majority of patients did not show specific radiological patterns. Cluster 3 showed more frequently an extrapulmonary cause of ARF, like septic shock (23% vs 7% and 2%), and also included more neutropenic patients compared to the overall population (38% vs 18% and 20%).

### Hospital mortality including clusters

**Fig. 3:**
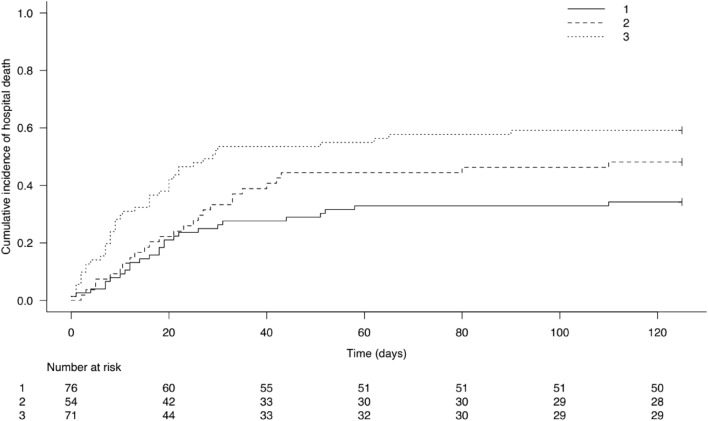
Cumulative incidence of hospital mortality according to the 3 clusters

As shown in Fig. 3, the cumulative incidence of hospital mortality was the highest for cluster 3 and lower for cluster 2 and cluster 1, and this difference was maintained over time.

After including the 3 clusters in the previous multivariate analysis, factors still associated with hospital mortality were leukemia-specific pulmonary involvement (OR = 5.07, 95% CI 1.95–13.18, *p* = 0.001), an ECOG Performance Status ≥ 2 (OR = 2.51, 95% CI 1.23–5.11, *p* = 0.01), a higher liver SOFA score (OR = 1.41, 95% CI 1.002–2.00, *p* = 0.049), as well as cluster 2 (OR = 2.48, 95% CI 1.03–6.01, *p* = 0.04) and cluster 3 (OR = 3.49, 95% CI 1.43–8.50, *p* = 0.006) compared to cluster 1. A focal alveolar chest X-ray pattern was still associated with a higher survival (OR = 0.17, 95% CI 0.07–0.46, *p* < 0.0001).

## Discussion

In this secondary analysis of the EFRAIM study focused on ARF in AML patients, we aimed to identify prognostic factors associated with poor outcomes. To do this, we pursued a two-step strategy that attempted to reproduce what happens at the patients ‘bedside.

In the first step, we reproduced the scenario in which clinicians face with single patients ‘characteristics, that require early recognition and early appropriate management because they could be associated with a worse outcome. Thus, we performed a parametric approach on clinical, biological and radiological variables and we compared them between survivors and not to hospital discharge.

One surprising finding was that the use of IMV was not associated with higher mortality in multivariate analysis, even if it was extensively performed. A possible explanation of this result could reside in timing, as median time from ARF onset to ICU admission was only 1 day and, if needed, IMV was performed early. In line with our hypothesis, Mokart et al. showed that a delay between respiratory symptoms’ onset and ICU management was associated with an adverse outcome in cancer patients [22]. Similarly, in a study on AML patients with ARF [6], IMV was strongly associated with mortality, but the median time to respiratory symptoms onset and ICU admission reached 3 or 4 days. These findings suggest that, when needed, IMV should occur early. In support of our results, Azoulay et al. described a 100% mortality rate in critically ill cancer patients with ARF if IMV was performed after the failure of NIV or > 72 h after ICU admission [23], while a meta-analysis by Dumas et al. showed that the time elapsed between ICU admission and intubation is a strong predictor of mortality in immunocompromised patients [24].

However, in our cohort there was no delay in ICU admission nor in performing intubation; these short timings, as well as the median PaO2/FiO2 ratio and the respiratory SOFA were comparable in survivors and non-survivors. Thus, when respiratory support is optimized, the difference in outcome might be due to other factors.

We found that the major predictors of hospital mortality were easily recognizable clinical characteristics at the time of ICU admission, namely the clinical presentation (respiratory symptoms and associated organ failures) and radiographic findings.

In multivariate analysis, the presence of cough, the most common among respiratory symptoms, was a strong predictor of hospital mortality; this data is even more interesting if we think that, in hematological patients, respiratory symptoms are more subtle than in the general population, and fever can be the only sign suggestive of underlying pneumonia. Furthermore, the only ARF etiology found to be independently associated with the presence of cough was viral infection. Generally, viruses are not the leading cause of ARF in this subset of patients [25]; nevertheless, in our cohort they accounted for up to 25% of ARF infective causes. These findings suggest that viruses can cause severe pneumonia in AML patients, and that cough could be a warning signal of an advanced pulmonary injury not to be underestimated [26].

Chest X-ray is one of the first line diagnostic strategy in hematological patients with neutropenic fever to rule out pulmonary involvement and is widely available in all care settings [25]. In our study, the presence of a focal alveolar pattern was associated with lower mortality, denoting that the extension of the lung injury can affect not only the clinical picture, but also outcome. As survivors and non-survivors had the same respiratory severity at ICU admission (as evidenced by similar values of respiratory SOFA score and PaO_2_/FiO_2_ ratio), the assessment of patient severity relied also on the radiological patterns, as a diffuse lung involvement had a worst prognosis than a localized one, and likely represent progression to acute respiratory distress syndrome (ARDS), in line with previous studies [27, 28].

In the second step, we assessed whether clinicians might be faced with syndromic subgroups of ARF phenotypes in AML patients, through a hierarchical cluster analysis. Hospital mortality, the main outcome, was not included among the variables analyzed; thus, it could not influence the result of our unsupervised analysis.

We identified three clusters of patients; the first one depicts a “leukemic cluster”, made up of AML patients admitted to ICU for the management of an isolate respiratory failure (apparently milder), in the presence of high-risk AML features, such as hyperleukocytosis; they showed a favorable outcome in 70% of cases, underscoring that leukemic pulmonary involvement is reversible if it responds to a promptly administered chemotherapy and an appropriate hematological management is provided. Furthermore, most patients in this cluster did not eventually require mechanical ventilation, fact that may have subsequently contributed to a better outcome, as previously reported by Heger et al. [29].

The second group is the “pulmonary cluster”, made up of severely immunocompromised patients who develop serious ARF, mostly of infectious origin; in this setting, ARF is characterized by diffuse lung injury requiring high oxygen support. Furthermore, as the time elapsed between ARF onset and ICU management was longer, a focal lung involvement might have widespread into diffuse and difficult-to-treat presentations. These patients were also more prone to viral infections, that frequently presented with cough, and ICU-acquired complications, as VAP, in line with previously reported studies [30]. This clinical scenario is well represented by the diffuse radiological patterns found in those patients: indeed, an extended radiological lung injury could be as severe as the need for vasopressors or the presence of other organ failures. Thus, it might be of interest to quantify the radiological pulmonary involvement both as an aetiologic determinant and a prognostic factor.

The third subphenotype is represented by the “clinical inflammatory cluster”, consisting of patients with multiorgan failures in addition to severe ARDS, requiring more critical care support. As the mortality rate was the highest compared to the two other clusters, the presence of multiple organ failures, especially hemodynamic instability, deserves special consideration and prompt intervention; indeed, despite the short delay between ARF onset and its ICU management, the treatment of other non-respiratory organ failures seemed to be delayed, as highlighted by the significantly higher non-respiratory SOFA scores at ICU admission recorded in this cluster compared to the other two.

When we entered the clusters in the original multivariate analysis, we found that the initial risk factors remained associated with a worse outcome, but also the pulmonary and the inflammatory clusters exerted an independent negative impact on survival.

Conversely, cough and the use of vasopressor were no longer present as independent variables, probably because they were strongly represented in cluster 2 and 3, respectively.

Thus, these clusters play a relevant prognostic role, but their identification could be also important in choosing an appropriate management strategy. For example, the leukemic cluster could benefit from an early chemotherapy initiation or from dexamethasone administration in case of higher WBC count, in order to lower early death rates [31]; on the same line, the inflammatory cluster may require a more aggressive ventilatory strategy. Indeed, Calfee et al. already described an hyperinflammatory ARDS phenotype similar to cluster 3, characterized by the use of vasopressor, metabolic acidosis and shock, in which the use of higher PEEP was associated with better outcomes [32].

One strength of this study is the multicenter international design; furthermore, the parameters that we find to be associated with outcome are easily and rapidly available in all care settings, making them reproducible to identify high-risk patients.

The major limitation of the study is the lack of detailed data on AML characteristics (i.e., FAB classification, karyotype, molecular abnormalities, ELN risk stratification), although these features were not associated with short-term survival in most reports [12–14, 33–35]; similarly, detailed information on HSCT features were not available, and thus could not be included in the analysis.

## Conclusions

Among AML patients with ARF, factors associated with a worse outcome are related to patient’s background (performance status, leukemic pulmonary involvement), symptoms, radiological findings, and timing, as the onset of other organ failures and a delay in ICU management can lead to very poor outcome.

We also identified 3 specific ARF syndromes in AML patients, which showed a prognostic significance and could guide clinicians to optimize management strategies.

### Supplementary Information

**Additional file 1.**

**Additional file 2: **Diagnostic workup and ARF etiology (univariate analysis).

**Additional file 3: **Variances of the first 5 dimensions.

**Additional file 4: **Variables’ contribution to the first dimension; B: variables’ contribution to the second dimension.

**Additional file 5: **A: quantitative variables’ contribution to the first and second dimensions. Quantitative variables are represented by arrows projecting lines (the arrows show the degree and the direction of contributions). B: qualitative variables’ contribution to the first and second dimensions. Qualitative variables are represented by triangles, reflecting variable’s centroid in the different levels of qualitative variables. The first dimension was mostly driven by the severity of organ failures such as SOFA score (with a major role played by the respiratory SOFA score), IMV, use of vasopressor, occurrence of septic shock and RRT. It was also associated with diffuse lung damage, like diffuse alveolar and ground glass patterns on CT-scan. The second dimension was mostly driven by respiratory parameters such as severe lung lesions on chest-Xray and CT-scan, including ground glass, alveolar and interstitial diffuse patterns, but also high respiratory rate.

**Additional file 6: **Clustering methodological design.

## Data Availability

The datasets used and/or analyzed during the current study are available from the corresponding author on reasonable request.
