# Supplementary material for: Characteristics and outcomes of patients with acute myeloid leukemia admitted to intensive care unit with acute respiratory failure: a post-hoc analysis of a prospective multicenter study
Source: Ann Intensive Care. 2023 Sep 2;13:79. doi: 10.1186/s13613-023-01172-3 (PMC10474995; doi:10.1186/s13613-023-01172-3)

Supplementary File 1

*Inclusion/exclusion criteria*

Inclusion criteria were age ≥18 years; a diagnosis of Acute Myeloid Leukemia (AML) active or in remission for ≤5 years, including acute promyelocytic leukemia; patients undergoing hematopoietic stem cell transplantation (HSCT) for AML; acute hypoxemic ARF (defined as labored breathing, respiratory distress, dyspnea at rest or cyanosis, or PaO2 <60 mmHg or SpO2 <90% on room air, or tachypnea >30/min with onset of respiratory symptoms <72h); need for oxygen ≥6 L/min [[1](#_ENREF_1)].

Exclusion criteria were admission to ICU after a cardiac arrest; ICU admission only for performing bronchoscopy; post-operative ARF and patient’s refusal to participate in the study. Patients were enrolled in the respect of local Research Ethics Board policies.

*Data collection*

Data collection included patients ’demographics and clinical characteristics at ICU admission [age, comorbidities, Eastern Cooperative Oncology Group (ECOG) performance status, SOFA score], respiratory symptoms and ventilatory values at admission and during the first 24h of ICU stay, as well as hematological features (disease status, HSCT, extramedullary disease). Patient’s code status on ICU admission was recorded as full code management, time-limited trial, early admission and do not intubate (DNI)/ do not resuscitate (DNR). Both non-invasive diagnostic strategies [(blood culture, sputa examination, viral PCR, echocardiography/lung ultrasound, chest X-ray and computerized tomography (CT) scan] and invasive test [fiberoptic bronchoscopy-bronchoalveolar lavage (FO-BAL), lung biopsy] were recorded. ARF etiologies included infectious, non-infectious (i.e., cardiogenic pulmonary oedema, disease-related infiltrate, drug toxicities) and undetermined causes. Oxygenation strategies [i.e., high-flow nasal cannula (HFNC), non-invasive ventilation (NIV), or invasive mechanical ventilation (IMV)] and critical care treatments [i.e., need for vasopressor or renal replacement therapy (RRT)] were recorded daily for the first 7 days of ICU stay and were managed by the attending physicians according to local ICU standard practice.

*Definitions*

AML was defined according to 2016 WHO classification [[2](#_ENREF_2)].

Leukemia-specific pulmonary involvement refers to either leukostasis, pulmonary infiltration by blasts or acute lysis pneumopathy [[3-5](#_ENREF_3)]. Leukostasis refers to the accumulation of blast cells in the lung vessels, usually associated with high leukemic cells count (> 50 x10⁹/L) and myelomonocytic (M4) or monocytic (M5) AML, as well as Fms-like tyrosine-3 (FLT3) mutated AML [[6](#_ENREF_6),[7](#_ENREF_7)]. The involved pathogenetic mechanisms could reside in increased blood viscosity, intravascular leukemic aggregation with microthrombi formation, endothelial cell damage or direct toxicity of the leukemic cells [[8](#_ENREF_8),[9](#_ENREF_9)], and it is associated with high mortality rate [[3](#_ENREF_3),[10](#_ENREF_10)].

Pulmonary infiltration by blast can result in lung infiltrates that typically follow the lymphatic routes along the bronchovascular bundles, pleural interstitial tissue and interlobular septa on computed tomography [[11](#_ENREF_11),[12](#_ENREF_12)]. It can occur with or without hyperleukocytosis, denoting that blast type shows different affinity for the pulmonary endothelium [[9](#_ENREF_9),[13](#_ENREF_13)].

Finally, acute lysis pneumopathy refers to respiratory deterioration after AML treatment initiation, mainly due to diffuse alveolar hemorrhage, as a manifestation of an acute tumor lysis syndrome [[14](#_ENREF_14),[15](#_ENREF_15)].

Neutropenia was defined as an absolute neutrophil count (ANC) of <500 cell/mm³ [[16](#_ENREF_16)].

Liver dysfunction was defined as a liver SOFA score ≥3; central nervous system (CNS) involvement was defined as neurological SOFA score ≥3.

*Identification of clusters*

A factor analysis of mixed data (FAMD) is a principal component method dedicated to analyzing complex datasets containing both quantitative and qualitative variables [[17](#_ENREF_17)]. This dimensions’ reduction algorithm summarizes the main variances of the variables and transposes them on lower dimensional planes, which allows simple visual evaluation of the data in a compact format; furthermore, it reduces the background noise of the variables and allows subsequent analysis, such as clustering. We did not enter hospital mortality in the model. Missing data were imputed by 2-dimensional FAMD model in order to reduce interpolation bias [[18](#_ENREF_18)].

Cluster analysis is one of the most popular unsupervised learning methods to identify subgroups sharing similar characteristics, with no need for predefined information. Thus, we performed an ascendant hierarchical cluster analysis (AHCA) on the dimensions provided by the FAMD model, using an ascendant algorithm on the Euclidean distances between points and according to the Ward’s method, which allows to minimize the total intracluster variance and generate the dendrogram. The clustering partition was obtained from hierarchical clustering and improved with the consolidation method [[19](#_ENREF_19)]. We identified the optimal number of clusters by the visual inspection of the dendrogram generated from the agglomerative hierarchical clustering.

*Factor analysis of mixed data (FAMD)*

FAMD was applied to the original dataset, which consisted of 75 clinical, radiological and biological parameters of the 201 AML patients with ARF. Eighty-seven dimensions were obtained. Variances of these 87 dimensions decreased gradually, and variances of the first 5 dimensions accounted for about 20% of the total variance. Thus, the first 5 dimensions were retained for further analysis (Supplementary File 3). The first two dimensions accounted for 5·2% and 4·1% of the variance, respectively. Variables’ contribution to these dimensions is shown in Supplementary File 4. Supplementary File 5 displays the two dimensions according to continuous and categorical variables.

*Ascendant hierarchical cluster analysis (AHCA)*

Then, hierarchical cluster analysis was performed with the matrix made with the values of the first 5 dimensions of 201 patients. A dendrogram showed the steps of cluster analysis. Three clusters were identified by visual inspection of inertia gain and by automatic selection (Figure 2, Supplementary File 6).

REFERENCES

1. Azoulay, E.; Pickkers, P.; Soares, M.; Perner, A.; Rello, J.; Bauer, P.R.; van de Louw, A.; Hemelaar, P.; Lemiale, V.; Taccone, F.S. Acute hypoxemic respiratory failure in immunocompromised patients: the Efraim multinational prospective cohort study. *Intensive care medicine* **2017**, *43*, 1808-1819.

2. Arber, D.A.; Orazi, A.; Hasserjian, R.; Thiele, J.; Borowitz, M.J.; Le Beau, M.M.; Bloomfield, C.D.; Cazzola, M.; Vardiman, J.W. The 2016 revision to the World Health Organization classification of myeloid neoplasms and acute leukemia. *Blood* **2016**, *127*, 2391-2405.

3. Moreau, A.-S.; Lengline, E.; Seguin, A.; Lemiale, V.; Canet, E.; Raffoux, E.; Schlemmer, B.; Azoulay, E. Respiratory events at the earliest phase of acute myeloid leukemia. *Leukemia & lymphoma* **2014**, *55*, 2556-2563.

4. Vincent, F. Leukostasis, infiltration and pulmonary lysis syndrome are the three patterns of leukemic pulmonary infiltrates. *Pulmonary Involvement in Patients with Hematological Malignancies* **2011**, 509-520.

5. Chaoui, D.; Legrand, O.; Roche, N.; Cornet, M.; Lefebvre, A.; Peffault de Latour, R.; Sanhes, L.; Huchon, G.; Marie, J.; Rabbat, A. Incidence and prognostic value of respiratory events in acute leukemia. *Leukemia* **2004**, *18*, 670-675.

6. Thornton, K.A.; Levis, M. FLT3 mutation and acute myelogenous leukemia with leukostasis. *New England Journal of Medicine* **2007**, *357*, 1639-1639.

7. Fröhling, S.; Schlenk, R.F.; Breitruck, J.; Benner, A.; Kreitmeier, S.; Tobis, K.; Döhner, H.; Döhner, K. Prognostic significance of activating FLT3 mutations in younger adults (16 to 60 years) with acute myeloid leukemia and normal cytogenetics: a study of the AML Study Group Ulm. *Blood, The Journal of the American Society of Hematology* **2002**, *100*, 4372-4380.

8. Würthner, J.U.; Köhler, G.; Behringer, D.; Lindemann, A.; Mertelsmann, R.; Lübbert, M. Leukostasis followed by hemorrhage complicating the initiation of chemotherapy in patients with acute myeloid leukemia and hyperleukocytosis: a clinicopathologic report of four cases. *Cancer: Interdisciplinary International Journal of the American Cancer Society* **1999**, *85*, 368-374.

9. Porcu, P.; Cripe, L.D.; Ng, E.W.; Bhatia, S.; Danielson, C.M.; Orazi, A.; McCarthy, L.J. Hyperleukocytic leukemias and leukostasis: a review of pathophysiology, clinical presentation and management. *Leukemia & lymphoma* **2000**, *39*, 1-18.

10. Lester, T.J.; Johnson, J.W.; Cuttner, J. Pulmonary leukostasis as the single worst prognostic factor in patients with acute myelocytic leukemia and hyperleukocytosis. *The American journal of medicine* **1985**, *79*, 43-48.

11. Prakash, U.B.; Divertie, M.B.; Banks, P.M. Aggressive therapy in acute respiratory failure from leukemic pulmonary infiltrates. *Chest* **1979**, *75*, 345-350.

12. Rossi, G.A.; Balbi, B.; Risso, M.; Repetto, M.; Ravazzoni, C. Acute myelomonocytic leukemia: demonstration of pulmonary involvement by bronchoalveolar lavage. *Chest* **1985**, *87*, 259-260.

13. Soares, F.A.; Landell, G.A.M.; Cardoso, M.C.d.M. Pulmonary leukostasis without hyperleukocytosis: a clinicopathologic study of 16 cases. *American journal of hematology* **1992**, *40*, 28-32.

14. Tryka, A.F.; Godleski, J.J.; Fanta, C.H. Leukemic cell lysis pneumonopathy a complication of treated myeloblastic leukemia. *Cancer* **1982**, *50*, 2763-2770.

15. Dombret, H.; Hunault, M.; Faucher, C.; Dombret, M.C.; Degos, L. Acute lysis pneumopathy after chemotherapy for acute myelomonocytic leukemia with abnormal marrow eosinophils. *Cancer* **1992**, *69*, 1356-1361.

16. Freifeld, A.G.; Bow, E.J.; Sepkowitz, K.A.; Boeckh, M.J.; Ito, J.I.; Mullen, C.A.; Raad, I.I.; Rolston, K.V.; Young, J.-A.H.; Wingard, J.R. Clinical practice guideline for the use of antimicrobial agents in neutropenic patients with cancer: 2010 update by the Infectious Diseases Society of America. *Clinical infectious diseases* **2011**, *52*, e56-e93.

17. Pagès, J. Factorial Analysis of Mixed Data. In *Multiple Factor Analysis by Example Using R*, Chapman and Hall/CRC: 2014; pp. 67-78.

18. Josse, J.; Husson, F. missMDA: a package for handling missing values in multivariate data analysis. *Journal of Statistical Software* **2016**, *70*, 1-31.

19. Husson, F.; Josse, J. Multivariate Data Analysis. In Proceedings of Special focus on clustering and multiway methods.[Internet document] https://www. rproject. org/conferences/useR-2010/tutorials/Husson+ Josse. pdf. Accessed; p. 2018.

Supplementary File 2. Diagnostic workup and ARF etiology (univariate analysis)

| **Characteristic** | **Overall (n=201)** | **Alive (n=107)** | **Dead (n=94)** | ***p* value** |
| --- | --- | --- | --- | --- |
| Positive diagnostic test, n (%) |  |  |  |  |
| Blood culture | 50 (24.9) | 32 (29.9) | 18 (19.1) | 0.11 |
| Bacteria | 47 (23.4) | 29 (27.1) | 18 (19.1) | 0.25 |
| Yeasts | 3 (1.5) | 3 (2.8) | 0 (0.0) | 0.29 |
| Sputum | 32 (15.9) | 19 (17.8) | 13 (13.8) | 0.57 |
| Induced sputum | 2 (1.0) | 1 (0.9) | 1 (1.1) | 1.00 |
| Nasopharyngeal aspirate | 27 (13.4) | 19 (17.8) | 8 (8.5) | 0.09 |
| Pleurocentesis | 2 (1.0) | 1 (0.9) | 1 (1.1) | 1.00 |
| Biomarkers | 99 (49.3) | 57 (53.3) | 42 (44.7) | 0.28 |
| CRP | 64 (31.8) | 39 (36.4) | 25 (26.6) | 0.18 |
| PCT | 54 (26.9) | 31 (29.0) | 23 (24.5) | 0.58 |
| NT-proBNP | 26 (12.9) | 14 (13.1) | 12 (12.8) | 1.00 |
| Antigenuria | 2 (1.0) | 1 (0.9) | 1 (1.1) | 1.00 |
| Serum GM | 12 (6.0) | 6 (5.6) | 6 (6.4) | 1.00 |
| Virus multiplex PCR | 40 (19.9) | 17 (15.9) | 23 (24.5) | 0.18 |
| Lung Ultrasound/Echo cardio | 42 (20.9) | 22 (20.6) | 20 (21.3) | 1.00 |
| Broncho-alveolar lavage, n (%) | 85 (42.3) | 41 (38.3) | 44 (46.8) | 0.28 |
| Lung biopsy, n (%) | 3 (1.5) | 0 (0.0) | 3 (3.2) | 0.20 |
|  |  |  |  |  |
| ARF etiology, n (%) |  |  |  |  |
| **Infectious** |  |  |  |  |
| Bacterial | 58 (28.8) | 35 (32.8) | 23 (24.5) | 0.39 |
| Clinically documented | 25 (12.4) | 16 (15.0) | 9 (9.6) |  |
| Micriobiologically documented | 33 (16.4) | 19 (17.8) | 14 (14.9) |  |
| *Gram+* | 15 (7.5) | 11 (10.3) | 4 (4.3) |  |
| *Gram-* | 18 (8.9) | 8 (7.5) | 10 (10.6) |  |
| Non bacterial | 143 (71.1) | 72 (67.3) | 71 (75.5) |  |
| Viral | 26 (12.9) | 11 (10.3) | 15 (16.0) | 0.32 |
| Influenza | 11 (5.5) | 4 (3.7) | 7 (7.4) | 0.40 |
| CMV | 2 (1.0) | 1 (0.9) | 1 (1.1) | 1.00 |
| RSV | 5 (2.5) | 2 (1.9) | 3 (3.2) | 0.88 |
| Other* | 8 (4.0) | 4 (3.7) | 4 (4.3) | 1.00 |
| HHV6 colonization/infection | 7 (3.5) | 5 (4.7) | 2 (2.1) | 0.55 |
| Invasive fungal infection | 19 (9.5) | 12 (11.2) | 7 (7.4) | 0.36 |
| IPA | 11 (5.5) | 7 (6.5) | 4 (4.3) | 0.69 |
| Candidemia | 6 (3.0) | 4 (3.7) | 2 (2.1) | 0.80 |
| *Pnuemocistis* pneumonia | 2 (1.0) | 1 (0.9) | 1 (1.1) | 1.00 |
| **Non-infectious** |  |  |  |  |
| Cardiogenic pulmonary edema | 13 (6.5) | 10 (9.3) | 3 (3.2) | 0.14 |
| Leukemic-specific PI | 35 (17.4) | 13 (12.1) | 22 (23.4) | 0.06 |
| Extrapulmonary | 22 (10.9) | 13 (12.1) | 9 (9.6) | 0.72 |
| Aspiration pneumonia | 5 (2.5) | 3 (2.8) | 2 (2.1) | 1.00 |
| Airway-related disorders | 3 (1.5) | 2 (1.9) | 1 (1.1) | 1.00 |
| Drug Toxicity | 9 (4.5) | 7 (6.5) | 2 (2.1) | 0.24 |
| Other** | 4 (2.0) | 3 (2.8) | 1 (1.1) | 0.62 |
| **Undetermined** | 28 (13.9) | 13 (12.1) | 15 (16.0) | 0.57 |
| More than one ARF etiology | 26 (12.9) | 18 (16.8) | 8 (8.5) | 0.12 |
| *CRP* C-reactive protein; *PCT* procalcitonin; *GM* galactomannan; *IPA* invasive pulmonary Aspergillosis; *PI* pulmonary involvement; Extrapulmonary: pulmonary repercussion of an extrapulmonary issue (i.e., desaturation during septic shock).  *: Coronavirus, Enterovirus, Human Parainfluenza Virus type 3, Rhinovirus  **: COPD, pleural involvement. | | | | |

Supplementary File 3: variances of the first 5 dimensions.

|  | Dim.1 | Dim.2 | Dim.3 | Dim.4 | Dim.5 |
| --- | --- | --- | --- | --- | --- |
| Variance | 4.482 | 3.533 | 3.014 | 2.901 | 2.732 |
| % of variance | 5.216 | 4.112 | 3.508 | 3.377 | 3.180 |
| Cumulative % of variance | 5.216 | 9.328 | 12.836 | 16.213 | 19.393 |
| *Dim* dimension | | | | | |

Supplementary File 4.A


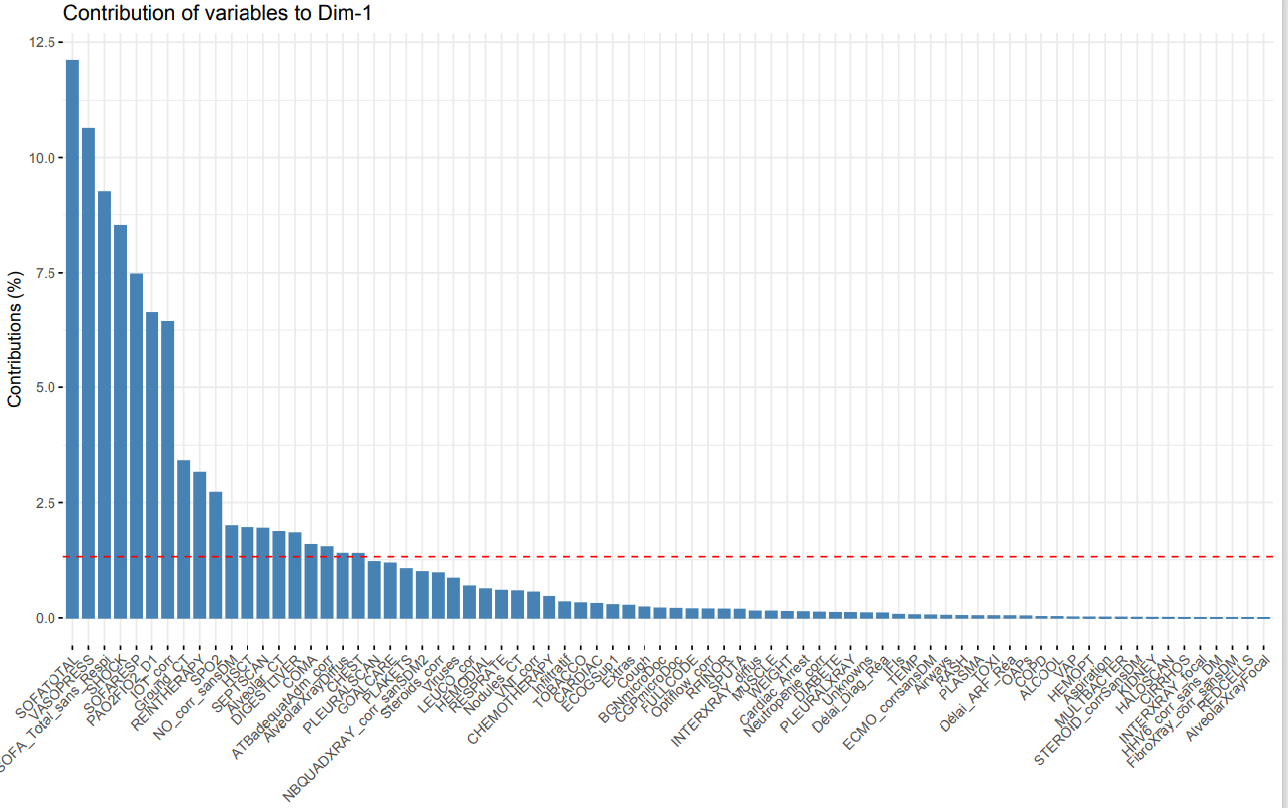


Supplementary File 4.B


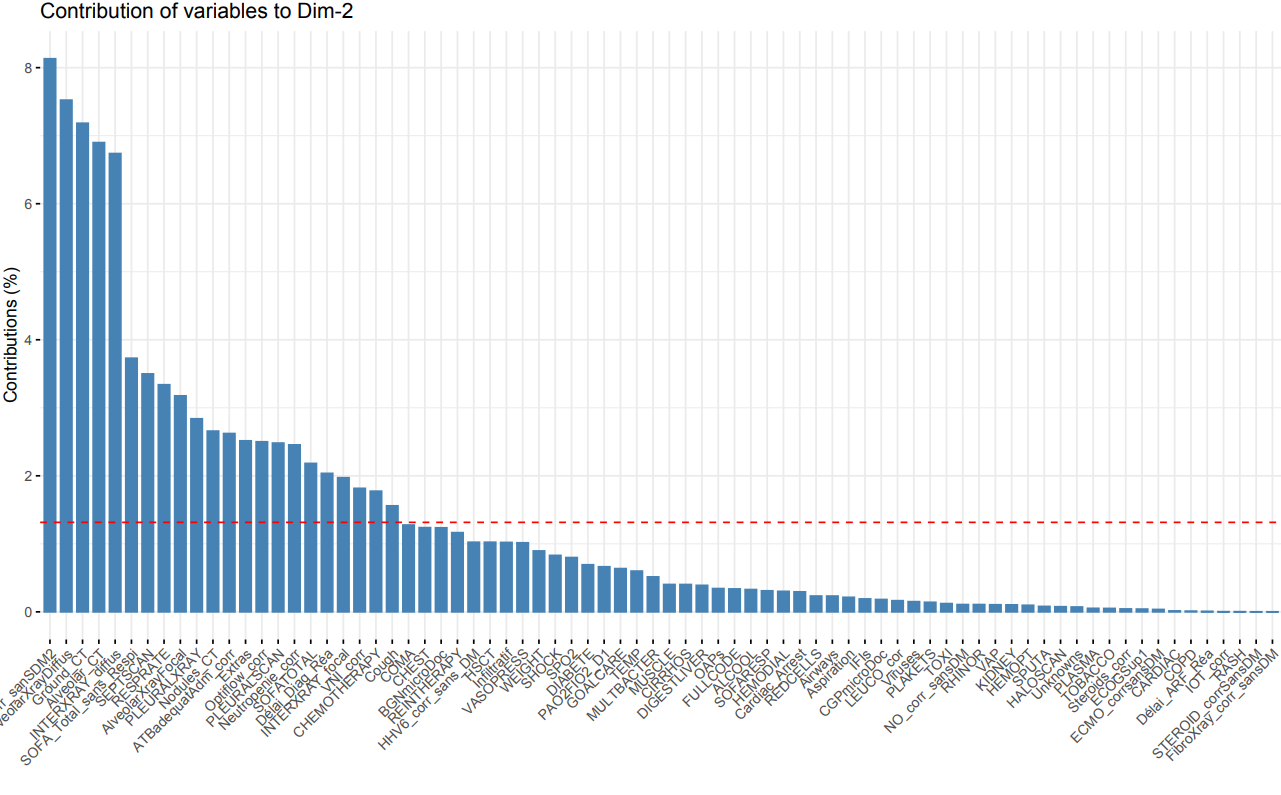


Supplementary File 4. A: variables’ contribution to the first dimension; B: variables’ contribution to the second dimension.

Supplementary File 5.A


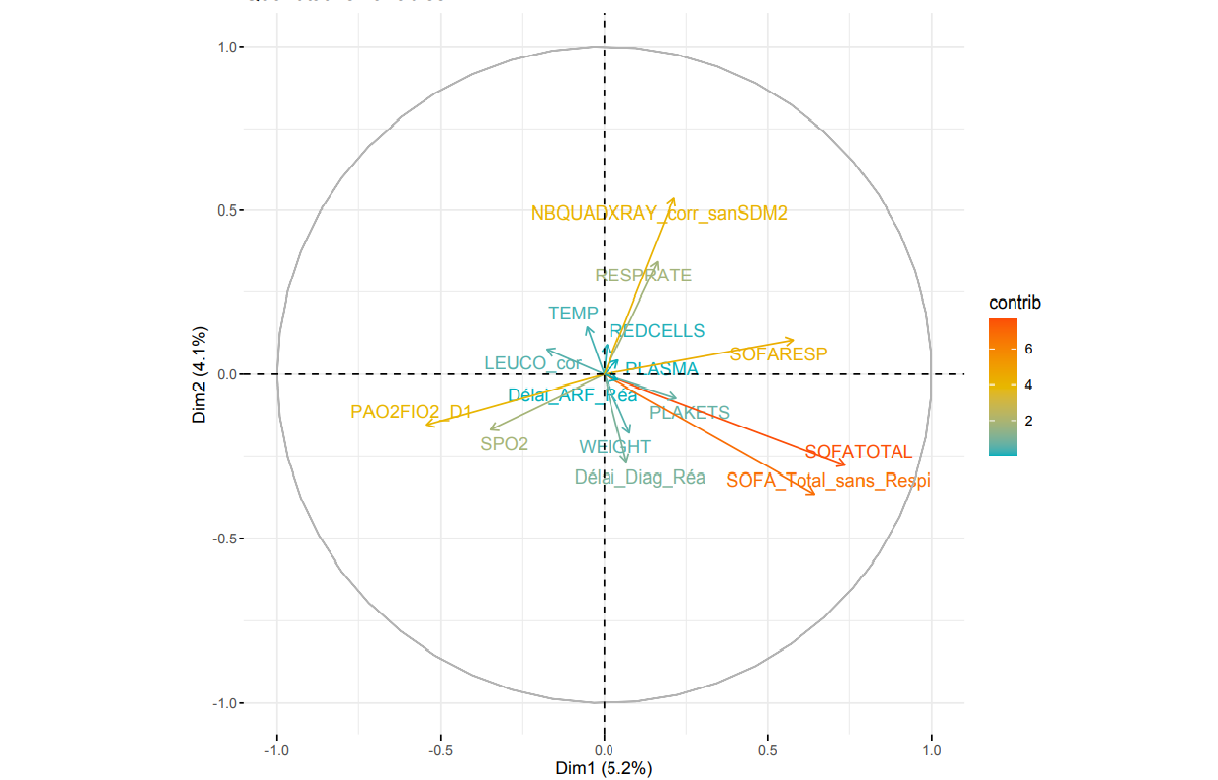


Supplementary File 5.B


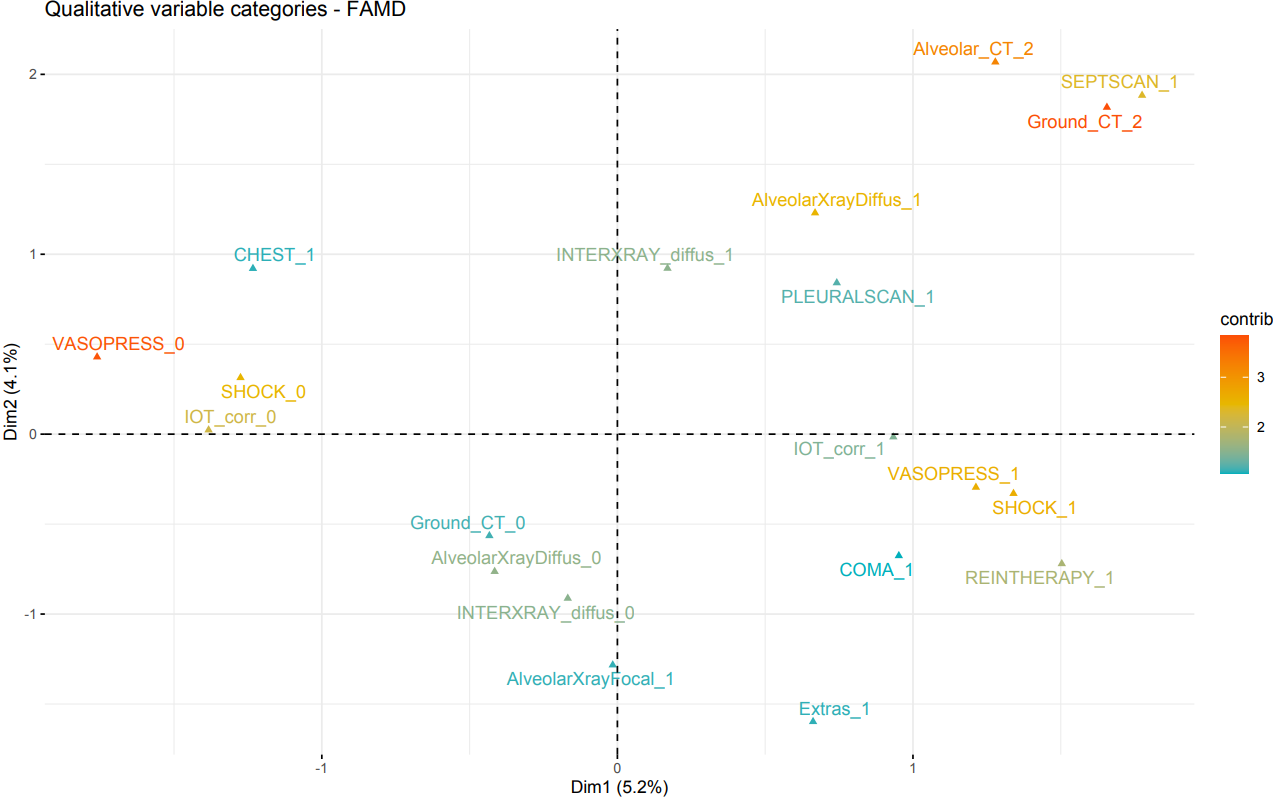


Supplementary File 5.A: quantitative variables’ contribution to the first and second dimensions. Quantitative variables are represented by arrows projecting lines (the arrows show the degree and the direction of contributions).

B: qualitative variables’ contribution to the first and second dimensions. Qualitative variables are represented by triangles, reflecting variable’s centroid in the different levels of qualitative variables.

The first dimension was mostly driven by the severity of organ failures such as SOFA score (with a major role played by the respiratory SOFA score), IMV, use of vasopressor, occurrence of septic shock and RRT. It was also associated with diffuse lung damage, like diffuse alveolar and ground glass patterns on CT-scan.

The second dimension was mostly driven by respiratory parameters such as severe lung lesions on chest-Xray and CT-scan, including ground glass, alveolar and interstitial diffuse patterns, but also high respiratory rate.

Supplementary File 6: clustering methodological design


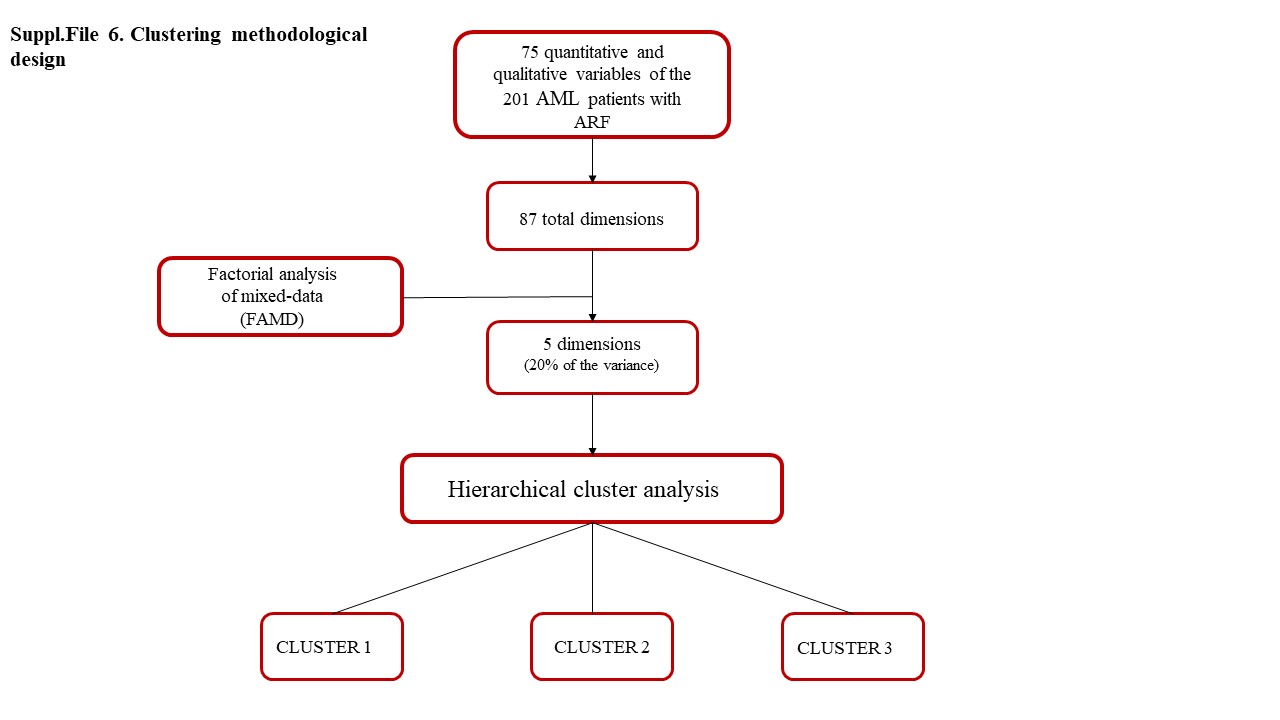

Supplement: Supplementary file 1 — Additional file 1. [file 13613_2023_1172_MOESM1_ESM.docx]
