# Supplementary material for: Characteristics and outcomes of patients with acute myeloid leukemia admitted to intensive care unit with acute respiratory failure: a post-hoc analysis of a prospective multicenter study
Source: Ann Intensive Care. 2023 Sep 2;13:79. doi: 10.1186/s13613-023-01172-3 (PMC10474995; doi:10.1186/s13613-023-01172-3)
Supplement: Supplementary file 2 — Additional file 2: Diagnostic workup and ARF etiology (univariate analysis). [file 13613_2023_1172_MOESM2_ESM.docx]

Additional File 2. Diagnostic workup and ARF etiology (univariate analysis)

| Characteristic | Overall (n=201) | Alive (n=107) | Dead (n=94) | *p* value |
| --- | --- | --- | --- | --- |
| Positive diagnostic test, n (%) |  |  |  |  |
| Blood culture | 50 (24·9) | 32 (29·9) | 18 (19·1) | 0·11 |
| Bacteria | 47 (23·4) | 29 (27·1) | 18 (19·1) | 0·25 |
| Yeasts | 3 (1·5) | 3 (2·8) | 0 (0·0) | 0·29 |
| Sputum | 32 (15·9) | 19 (17·8) | 13 (13·8) | 0·57 |
| Induced sputum | 2 (1·0) | 1 (0·9) | 1 (1·1) | 1·00 |
| Nasopharyngeal aspirate | 27 (13·4) | 19 (17·8) | 8 (8·5) | 0·09 |
| Pleurocentesis | 2 (1·0) | 1 (0·9) | 1 (1·1) | 1·00 |
| Biomarkers | 99 (49·3) | 57 (53·3) | 42 (44·7) | 0·28 |
| CRP | 64 (31·8) | 39 (36·4) | 25 (26·6) | 0·18 |
| PCT | 54 (26·9) | 31 (29·0) | 23 (24·5) | 0·58 |
| NT-proBNP | 26 (12·9) | 14 (13·1) | 12 (12·8) | 1·00 |
| Antigenuria | 2 (1·0) | 1 (0·9) | 1 (1·1) | 1·00 |
| Serum GM | 12 (6·0) | 6 (5·6) | 6 (6·4) | 1·00 |
| Virus multiplex PCR | 40 (19·9) | 17 (15·9) | 23 (24·5) | 0·18 |
| Lung Ultrasound/Echo cardio | 42 (20·9) | 22 (20·6) | 20 (21·3) | 1·00 |
| Broncho-alveolar lavage, n (%) | 85 (42·3) | 41 (38·3) | 44 (46·8) | 0·28 |
| Lung biopsy, n (%) | 3 (1·5) | 0 (0·0) | 3 (3·2) | 0·20 |
|  |  |  |  |  |
| ARF etiology, n (%) |  |  |  |  |
| **Infectious** |  |  |  |  |
| Bacterial | 58 (28·8) | 35 (32·8) | 23 (24·5) | 0·39 |
| Clinically documented | 25 (12·4) | 16 (15·0) | 9 (9·6) |  |
| Micriobiologically documented | 33 (16·4) | 19 (17·8) | 14 (14·9) |  |
| *Gram+* | 15 (7·5) | 11 (10·3) | 4 (4·3) |  |
| *Gram-* | 18 (8·9) | 8 (7·5) | 10 (10·6) |  |
| Non bacterial | 143 (71·1) | 72 (67·3) | 71 (75·5) |  |
| Viral | 26 (12·9) | 11 (10·3) | 15 (16·0) | 0·32 |
| Influenza | 11 (5·5) | 4 (3·7) | 7 (7·4) | 0·40 |
| CMV | 2 (1·0) | 1 (0·9) | 1 (1·1) | 1·00 |
| RSV | 5 (2·5) | 2 (1·9) | 3 (3·2) | 0·88 |
| Other* | 8 (4·0) | 4 (3·7) | 4 (4·3) | 1·00 |
| HHV6 colonization/infection | 7 (3·5) | 5 (4·7) | 2 (2·1) | 0·55 |
| Invasive fungal infection | 19 (9·5) | 12 (11·2) | 7 (7·4) | 0·36 |
| IPA | 11 (5·5) | 7 (6·5) | 4 (4·3) | 0·69 |
| Candidemia | 6 (3·0) | 4 (3·7) | 2 (2·1) | 0·80 |
| *Pnuemocistis* pneumonia | 2 (1·0) | 1 (0·9) | 1 (1·1) | 1·00 |
| **Non-infectious** |  |  |  |  |
| Cardiogenic pulmonary edema | 13 (6·5) | 10 (9·3) | 3 (3·2) | 0·14 |
| Leukemic-specific PI | 35 (17·4) | 13 (12·1) | 22 (23·4) | 0·06 |
| Extrapulmonary | 22 (10·9) | 13 (12·1) | 9 (9·6) | 0·72 |
| Aspiration pneumonia | 5 (2·5) | 3 (2·8) | 2 (2·1) | 1·00 |
| Airway-related disorders | 3 (1·5) | 2 (1·9) | 1 (1·1) | 1·00 |
| Drug Toxicity | 9 (4·5) | 7 (6·5) | 2 (2·1) | 0·24 |
| Other** | 4 (2·0) | 3 (2·8) | 1 (1·1) | 0·62 |
| **Undetermined** | 28 (13·9) | 13 (12·1) | 15 (16·0) | 0·57 |
| More than one ARF etiology | 26 (12·9) | 18 (16·8) | 8 (8·5) | 0·12 |
|  |  |  |  |  |

CRP: C-reactive protein; PCT: procalcitonin; GM: galactomannan; IPA: invasive pulmonary Aspergillosis; PI: pulmonary involvement; Extrapulmonary: pulmonary repercussion of an extrapulmonary issue (i.e., desaturation during septic shock).

*: Coronavirus, Enterovirus, Human Parainfluenza Virus type 3, Rhinovirus

**: COPD, pleural involvement.
