# Supplementary material for: Characteristics and outcomes of patients with acute myeloid leukemia admitted to intensive care unit with acute respiratory failure: a post-hoc analysis of a prospective multicenter study
Source: Ann Intensive Care. 2023 Sep 2;13:79. doi: 10.1186/s13613-023-01172-3 (PMC10474995; doi:10.1186/s13613-023-01172-3)
Supplement: Supplementary file 3 — Additional file 3: Variances of the first 5 dimensions. [file 13613_2023_1172_MOESM3_ESM.docx]

Additional File 3: variances of the first 5 dimensions.

|  | Dim.1 | Dim.2 | Dim.3 | Dim.4 | Dim.5 |
| --- | --- | --- | --- | --- | --- |
|  |  |  |  |  |  |
|  |  |  |  |  |  |
| Variance | 4·482 | 3·533 | 3·014 | 2·901 | 2·732 |
| % of variance | 5·216 | 4·112 | 3·508 | 3·377 | 3·180 |
| Cumulative % of variance | 5·216 | 9·328 | 12·836 | 16·213 | 19·393 |
|  |  |  |  |  |  |

Dim.: dimension
