# Supplementary material for: Characteristics and outcomes of patients with acute myeloid leukemia admitted to intensive care unit with acute respiratory failure: a post-hoc analysis of a prospective multicenter study
Source: Ann Intensive Care. 2023 Sep 2;13:79. doi: 10.1186/s13613-023-01172-3 (PMC10474995; doi:10.1186/s13613-023-01172-3)
Supplement: Supplementary file 4 — Additional file 4: Variables’ contribution to the first dimension; B: variables’ contribution to the second dimension. [file 13613_2023_1172_MOESM4_ESM.docx]

Additional File 4.A


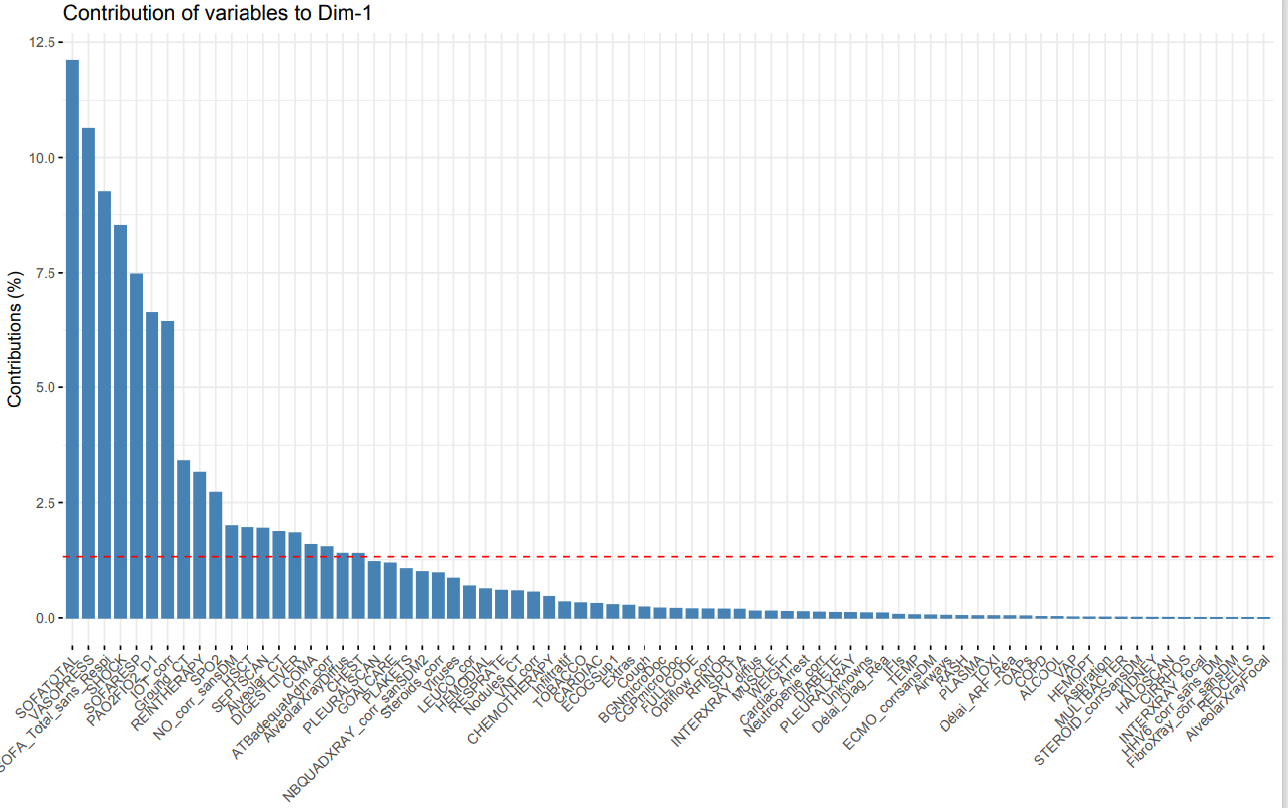


Additional File 4.B


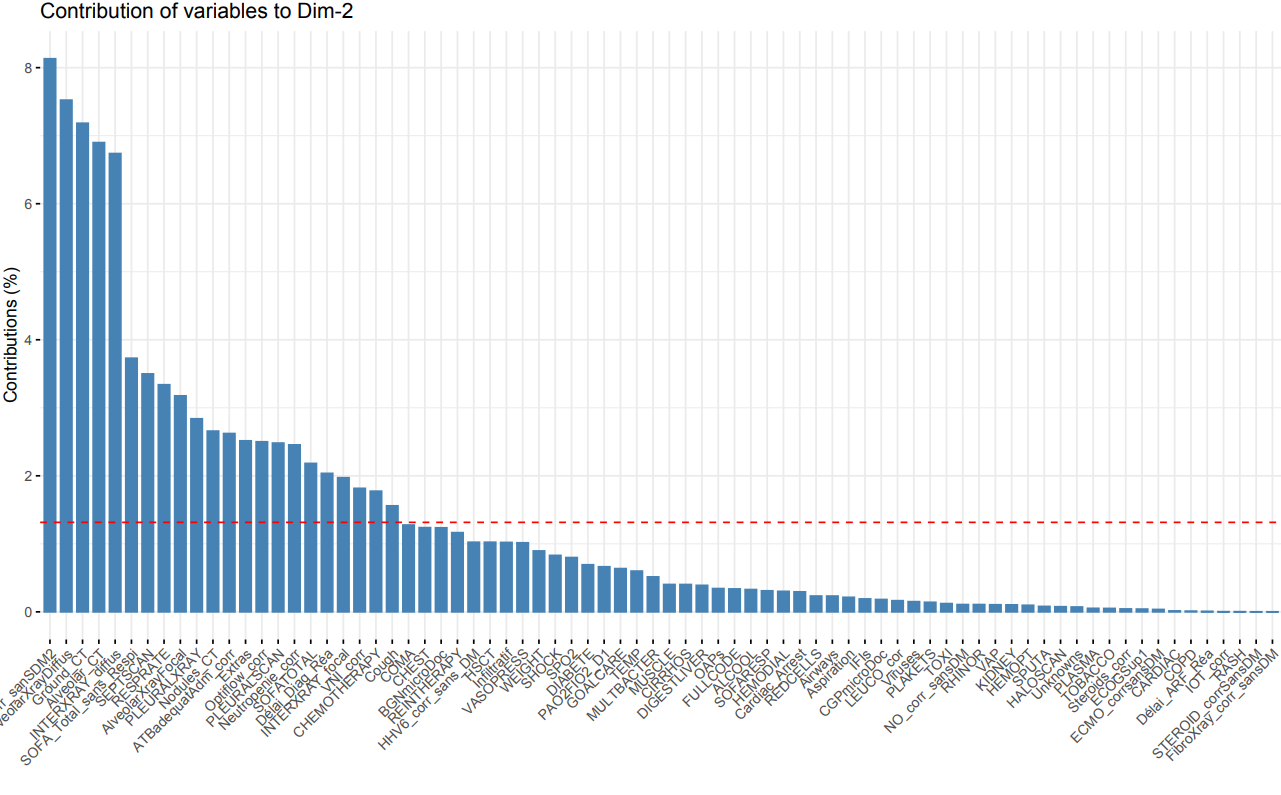


Additional File 4. A: variables’ contribution to the first dimension; B: variables’ contribution to the second dimension.
