# Supplementary material for: Characteristics and outcomes of patients with acute myeloid leukemia admitted to intensive care unit with acute respiratory failure: a post-hoc analysis of a prospective multicenter study
Source: Ann Intensive Care. 2023 Sep 2;13:79. doi: 10.1186/s13613-023-01172-3 (PMC10474995; doi:10.1186/s13613-023-01172-3)
Supplement: Supplementary file 5 — Additional file 5: A: quantitative variables’ contribution to the first and second dimensions. Quantitative variables are represented by arrows projecting lines (the arrows show the degree and the direction of contributions). B: qualitative variables’ contribution to the first and second dimensions. Qualitative variables are represented by triangles, reflecting variable’s centroid in the different levels of qualitative variables. The first dimension was mostly driven by the severity of organ failures such as SOFA score (with a major role played by the respiratory SOFA score), IMV, use of vasopressor, occurrence of septic shock and RRT. It was also associated with diffuse lung damage, like diffuse alveolar and ground glass patterns on CT-scan. The second dimension was mostly driven by respiratory parameters such as severe lung lesions on chest-Xray and CT-scan, including ground glass, alveolar and interstitial diffuse patterns, but also high respiratory rate. [file 13613_2023_1172_MOESM5_ESM.docx]

Additional File 5.A


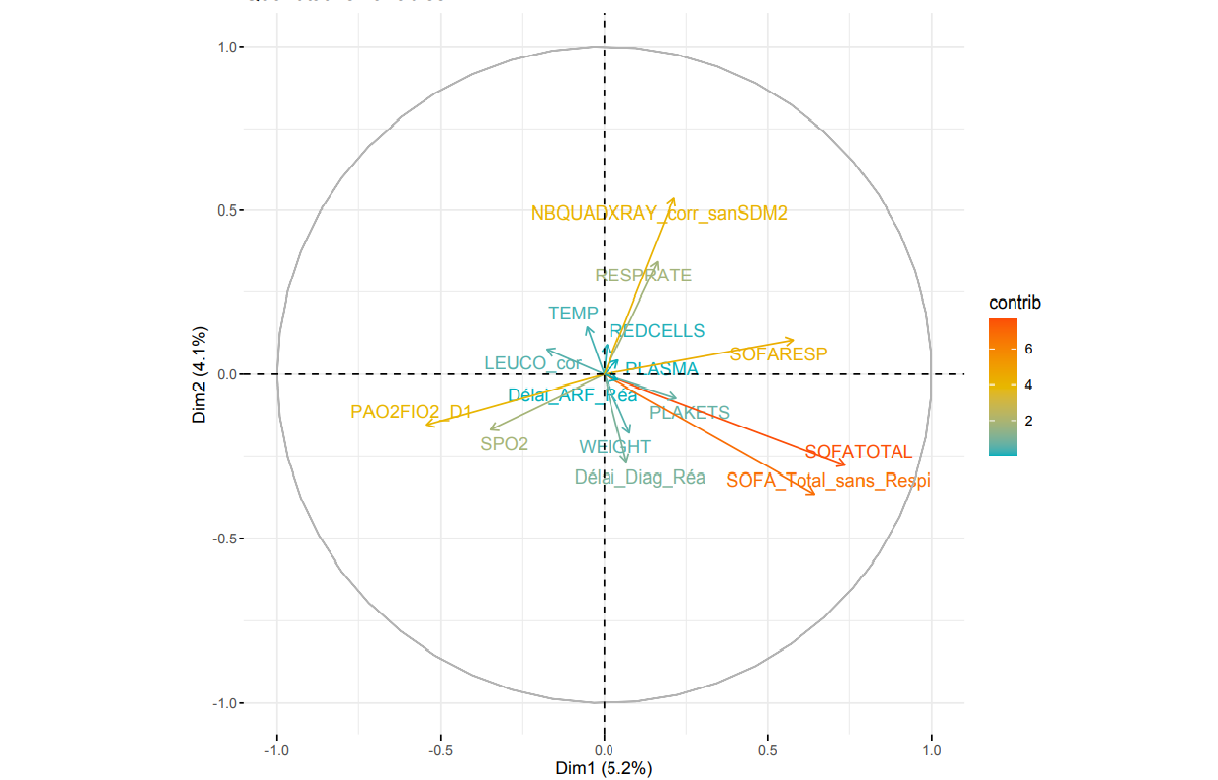


Additional File 5.B


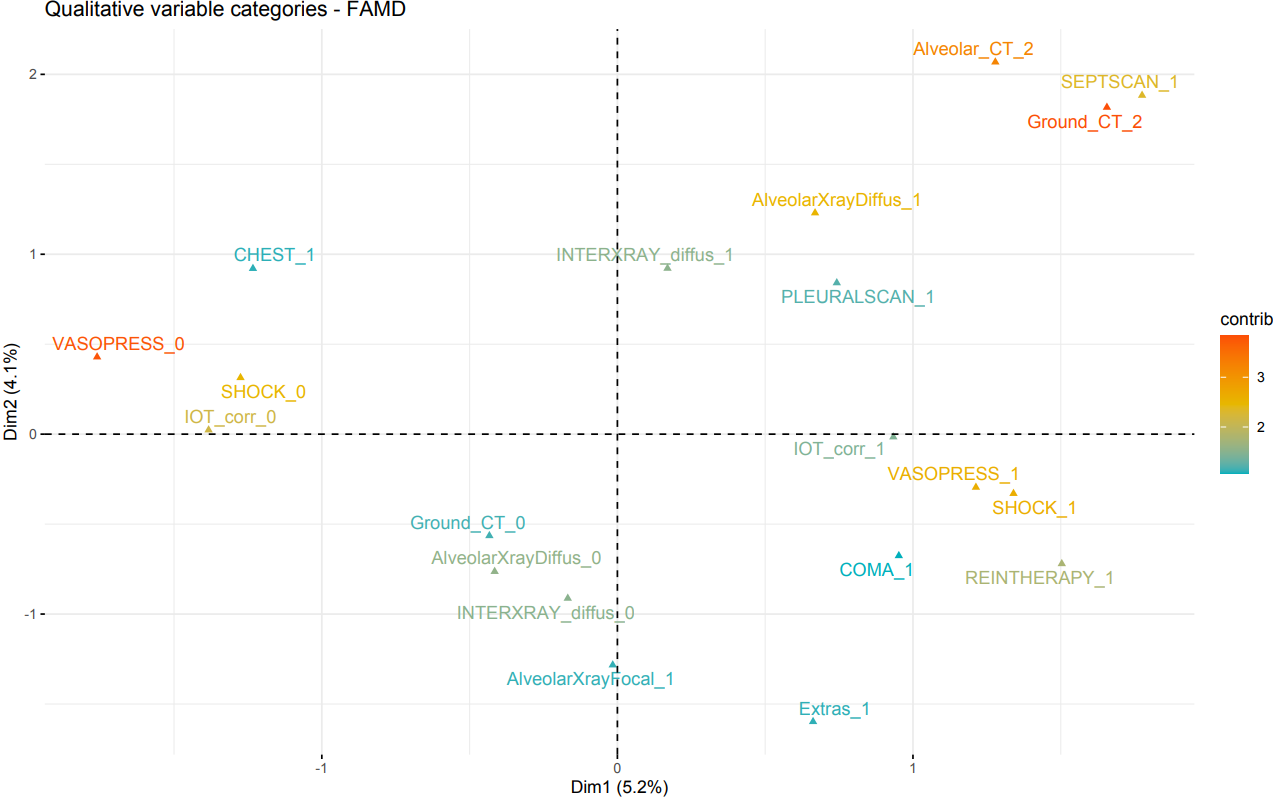


Additional File 5.A: quantitative variables’ contribution to the first and second dimensions. Quantitative variables are represented by arrows projecting lines (the arrows show the degree and the direction of contributions).

B: qualitative variables’ contribution to the first and second dimensions. Qualitative variables are represented by triangles, reflecting variable’s centroid in the different levels of qualitative variables.

The first dimension was mostly driven by the severity of organ failures such as SOFA score (with a major role played by the respiratory SOFA score), IMV, use of vasopressor, occurrence of septic shock and RRT. It was also associated with diffuse lung damage, like diffuse alveolar and ground glass patterns on CT-scan.

The second dimension was mostly driven by respiratory parameters such as severe lung lesions on chest-Xray and CT-scan, including ground glass, alveolar and interstitial diffuse patterns, but also high respiratory rate.
