# Supplementary figures and images for: Characteristics and outcomes of patients with acute myeloid leukemia admitted to intensive care unit with acute respiratory failure: a post-hoc analysis of a prospective multicenter study
Source: Ann Intensive Care. 2023 Sep 2;13:79. doi: 10.1186/s13613-023-01172-3 (PMC10474995; doi:10.1186/s13613-023-01172-3)

Additional File 6: clustering methodological design


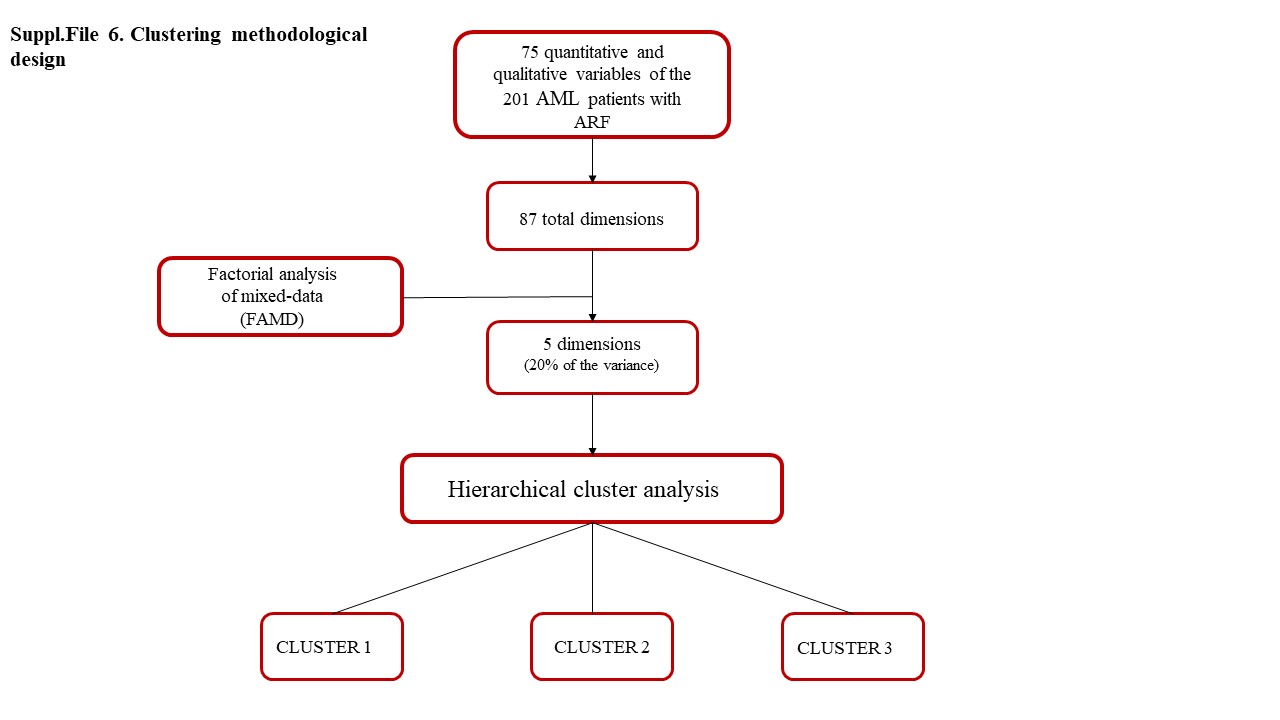

Supplement: Supplementary file 6 — Additional file 6: Clustering methodological design. [file 13613_2023_1172_MOESM6_ESM.docx]
